# Leaching-Resistant Self-Plasticized Poly(Vinyl Chloride) (PVC) as a Matrix for Ion-Selective Electrodes

**DOI:** 10.1021/acsmeasuresciau.6c00163

**Published:** 2026-07-26

**Authors:** Geetansh Gupta, Tara Forrest, Eric Bakker

**Affiliations:** Department of Inorganic and Analytical Chemistry, 27212 University of Geneva, Quai Ernest-Ansermet 30, Geneva CH-1211, Switzerland

**Keywords:** polymer, ion-selective electrode, click chemistry, leaching, self-plasticization

## Abstract

Polymer-based ion-selective electrodes typically rely on plasticized polymeric membranes to incorporate functional sensing components, but leaching of plasticizer into solution can negatively affect the response stability, operability, and overall lifetime of these sensors. Moreover, plasticizer loss can result in cytotoxicity, which is especially undesirable in wearable sensors and bioanalytical probes, requiring strict regulation and monitoring of the plasticizers used. To help overcome this challenge, a new class of self-plasticized poly­(vinyl chloride) (PVC) derivatives was synthesized here by grafting a plasticizer-like group onto azide-modified PVC using a highly efficient azide–alkyne Huisgen cycloaddition “click” reaction. The resulting polymer is naturally elastic and malleable that allows for the dissolution of sensing components and ion diffusion through the selective membrane, overcoming the need for an additional plasticizer. When applied to a library of ions (Ca^2+^, H^+^, K^+^, NO_3_^–^), the resulting membrane electrodes demonstrated promising stability and lifetime with an overall drift of 0.3 mV for the calcium-selective electrode over a testing period of 5 days. This synthetic approach provides a promising pathway for developing plasticizer leak-free, high-performance, and operationally stable sensors.

## Graphical abstract

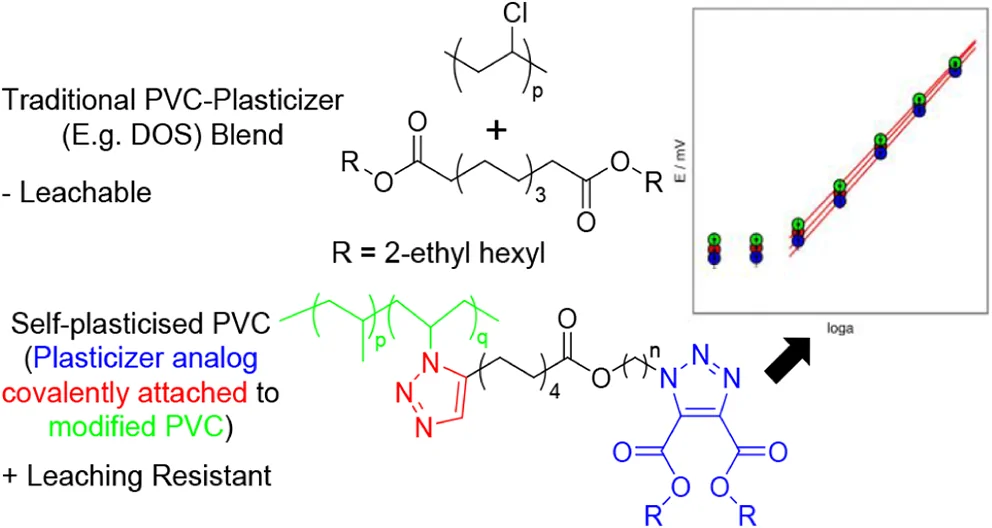

## Introduction

Poly­(vinyl chloride) (PVC) is a polymeric material widely used in different industries such as healthcare, construction, electronics, and daily consumer products. Owing to its rigidity and thermal instability at higher temperatures,^1^ nonvolatile organic liquids called plasticizers are routinely added to the polymer to make it softer and more malleable by reducing the glass transition temperature.^1^ Typical plasticizers used today include phthalates (e.g., bis­(2-ethylhexyl) phthalate—DEHP), adipates (e.g., dioctyl adipate—DOA), sebacates (e.g., dioctyl sebacate—DOS), or trimellitates (e.g., trioctyl trimellitate—TOTM). A major drawback of these polymer-plasticizer systems is the diffusional loss of plasticizer from these blends by a process called leaching, not only affecting the properties of the polymer but also raising concerns for the environment, wildlife, and human life.^2,3^ This is especially concerning for medical devices based on PVC, where plasticizer leachates may directly enter the human body via body fluids such as blood.^4–6^ Phthalate-based plasticizers such as bis­(2-ethylhexyl) phthalate (DEHP), di-*n*-butyl phthalate (DBP), or diisodecyl phthalate (DIDP) have been under intense scrutiny due to their prevalence in commonly encountered products such as personal care items, toys, or food packaging,^7^ and their potential carcinogenicity and ability to disrupt endocrine systems.^3,8^

To overcome these limitations and concerns, biobased and sustainable plasticizers have become potential eco-friendly options. These include epoxidized vegetable oils from soybean and linseed,^9^ citrate esters (e.g., acetyl tributyl citrate),^10^ and cardanol derivatives from cashew nuts.^11^ However, they fail to resolve the fundamental issue of leaching, making them unsuitable for medical- and food-grade applications. Alternate strategies such as limiting the movement of plasticizers by applying surface coatings^4,12^ have been effective but often lead to increased production costs and manufacturing complexities. Other methods such as the use of polymeric plasticizers,^13^ small branched plasticizer analogues,^14^ or ionic liquids^15^ have shown promise in slowing the depletion of additives; however, none of these solutions fully eliminate the migration concerns inherent in traditional PVC materials.

Polymer-based ion-selective membranes are an important type and component of electrochemical sensors routinely used in analytical measurements across fields such as environmental analysis and clinical and pharmaceutical analysis. These membranes have also been based on plasticized PVC to generate soft and flexible membranes that can be doped with various important sensing components such as ionophores^16^ and ion-exchangers.^17,18^ Depending on their chemical structures, these membrane components can also be prone to leaching.^19,20^ The effects of this leaching process on the lifetime and operation of the ion-selective membranes have been well studied in the field.^21^ An eventual loss of selectivity, sensitivity, and stable responses is observed, with concerns of cytotoxicity in the usage of sensors designed for *in vivo* analysis. Cosofret et al. already demonstrated in 1994 that plasticizer diffusing out of the membrane induces an increase in total leukocytes, resulting in a potential inflammatory response.^22,23^ These early findings were later supported by studies performed on mouse cell lines^24,25^ and human cell lines.^26^

To overcome the challenge of leaching of membrane components, various strategies have been developed and studied. A popular approach involves the use of a methacrylate-type or methacrylic–acrylic-type copolymer. The amount of each monomer can be adjusted during the synthesis to create a matrix that has a subzero glass transition temperature, surpassing the need for an additional plasticizer.^27,28^ This approach along with light-activated polymerization or photocuring^29^ was also utilized to tether the copolymer and ionophore to the matrix. While this improved stability,^30–34^ suboptimal reproducibility was observed owing to changes in polymer properties as a result of small variations during synthesis. Additionally, exposure to light may promote the degradation of light-sensitive membrane components within the matrix.^35^ Utilizing this approach, Qin and Bakker in 2003 demonstrated an example of a fully immobilized system by covalently anchoring a dodecacarborane anion to a methyl methacrylate–decyl methacrylate backbone, effectively serving as a cation exchanger.^36^ Subsequent developments by Bühlmann and coworkers yielded a pH-selective electrode featuring either the ionophore or the cation exchanger tethered to the copolymer;^37^ however, the attachment of both the components together resulted in a loss of functionality of the sensor. Similarly, this approach was applied to optical sensors (optodes), where the copolymer was used as a sensing matrix^38^ or as a backbone with chromoionophores grafted to it.^35^ Notably, the latter application revealed limitations in self-plasticized poly­(*n*-butyl acrylate) matrices, as the copolymerization process between the *n*-butyl acrylate monomer and the modified chromoionophore yielded nonfunctional sensors.

Despite the promising performance of methacrylate-based polymers, they are chemically different from classical PVC-based membranes more commonly utilized in the fabrication of ion-selective membranes. To remain closer to the classical system, poly­(vinylidene chloride) was tested in ion-selective membranes without plasticizer and gave satisfying results with an extended lifetime^39^ while being structurally similar to PVC. Direct immobilization of ionophores onto the PVC backbone has also been explored with carboxylated PVC.^40^ Similarly, azide-modified PVC,^41^ synthesized via click chemistry, has also been utilized. This approach, in particular, is appealing owing to its simplicity, minimal cross-reactivity, and high yields. Our group reported on azide-modified PVC where click chemistry was used to attach fluorescent molecules to the surface of an ion-selective membrane^42^ or poly­(ethylene glycol) (PEG) chains to enhance biocompatibility.^43^ More recently, we also investigated the use of click chemistry to tune the surface properties of an azide-bearing transducing layer.^44^

As an alternative, the chemical modification of PVC to enhance flexibility and malleability has been explored through various pathways, such as cross-linking.^45–47^ Although this approach successfully suppressed plasticizer leakage, it often introduced complications by altering the polymer’s fundamental physicochemical properties, such as increasing viscosity or compromising thermal stability.^48^ However, a more effective strategy by Navarro and coworkers involves grafting a thiol-modified DEHP derivative to the PVC backbone to produce an internally plasticized polymer.^49^ Adapting a similar approach, the substitution of chlorine groups in PVC with azide groups opened a pathway for performing click chemistry in which an azide-modified polymer can react with an alkyne-bearing plasticizer-like compound.

Outside the field of sensor development, significant contributions have been provided by Braslau and coworkers by taking advantage of the triazole ring formation during click reactions to generate immobilized phthalate analogues acting as plasticizers.^50^ While this initial approach failed to reach the desired range of glass transition temperatures, further work employed alkyl chain linkers to distance the plasticizing moiety from the polymer backbone alongside various ester substitutions on the alkyne precursor. With this new approach, a broad library of polymers was produced with glass transition values ranging from 96 °C down to −42 °C, which is similar to that of the plasticized PVC, thus effectively eliminating plasticizer leaching.^51^ Adapting a similar procedure, Deng et al. synthesized an internally plasticized PVC with a castor oil derivative.^52^

In this work, we present the synthesis of a novel class of self-plasticized PVC polymer derivatives as a matrix for ion-selective membranes. By utilizing click chemistry, a plasticizer analogue was grafted onto the modified PVC backbone that forms a flexible, elastic polymer, surpassing the need for an additional plasticizer to form membranes. The applicability of this polymer material for solid-contact ion-selective electrodes was demonstrated by testing a library of membranes for the detection of various ions (Ca^2+^, H^+^, K^+^, NO_3_^–^). This synthetic approach holds the potential to enable the development of plasticizer leak-free, high-performance, operationally stable sensors.

## Experimental Section

### Materials and Reagents

All aqueous solutions were prepared in deionized water (>18 MΩ cm). Poly­(vinyl chloride) (Selectophore, PVC), sodium azide (≥99.5%, NaN_3_), *para-*toluenesulfonic acid monohydrate (≥98.5%, *p*TSA), sodium chloride (≥99.5%, NaCl), sodium bromide (≥99.0%, NaBr), sodium sulfate (≥99.5%, Na_2_SO_4_), sodium nitrate (≥99.0%, NaNO_3_), potassium chloride (≥99.5%, KCl), lithium chloride (≥99.0%, LiCl), sodium bicarbonate (≥99.5%, NaHCO_3_), magnesium chloride hexahydrate (≥99.0%, MgCl_2_), calcium chloride (≥97.0%, CaCl_2_), 3-azido-1-propanol (≥96.0%), 10-undecynoic acid (95.0%), copper­(II) sulfate pentahydrate (≥96.0%), l-ascorbic acid (≥98.0%), sodium tetrakis­[3,5-bis­(trifluoromethyl)­phenyl]­borate (≥95.0%, Selectophore, NaTFPB), valinomycin (Selectophore, K-ionophore­(I)), ETH 5234 (Selectophore, calcium ionophore (IV)), tridodecylamine (Selectophore, H-ionophore­(I)), tridodecylmethylammonium nitrate (≥99,0%, TDMAN), bis­(2-ethylhexyl) phthalate (Selectophore, DEHP), phosphoric acid (≥85.0 wt %, H_3_PO_4_), and tetrahydrofuran (≥99.5%, Selectophore, THF) were purchased from Sigma-Aldrich.

Laboratory reagent grade toluene, analytical reagent grade chloroform (≥99.8%, CHCl_3_), laboratory reagent grade ethyl acetate (≥99.0%, EtOAc), and analytical reagent grade tetrahydrofuran (≥99.8%, THF) were purchased from Fisher Scientific.

Anhydrous magnesium sulfate (≥98.0%, MgSO_4_) was purchased from Santa Cruz Biotechnology. 2-Ethyl-1-hexanol (≥99.0%) was purchased from Fluka Analytical. Acetylenedicarboxylic acid (97%) was purchased from abcr. 6-azido-1-hexanol (≥96.0%) was purchased from Fluorochem. 10-Azido-1-decanol (≥95.0%) was purchased from abcr. Methanol (99.0%, MeOH) was purchased from Thommen-Furler AG. Citric acid monohydrate (99.5%) and boric acid (99.5%, H_3_BO_3_) were purchased from Acros Organics. *N*,*N*-Dimethylformamide (99.5%, DMF) and standardized sodium hydroxide (1.0 N, NaOH) were purchased from Honeywell-Fluka. Potassium tetrakis­(pentafluorophenyl)­borate (≥97%, KTPFPB) was purchased from Alfa Aesar. HPLC-grade acetonitrile (≥99.5%, ACN) and HPLC-grade *n*-hexane (97.0%) were purchased from Fluka Analytical. Single-walled octadecylamine-functionalized carbon nanotubes (fCNTs) were purchased from Sigma-Aldrich.

### Laboratory Instruments

Glassy carbon (GC) electrodes fabricated by encasing a GC rod (Ø 2.00 mm) in a polyether ether ketone (PEEK) tube were polished using different diamond powder suspensions (Ø 6, 3, 1, and 0.25 μm) prior to use. Impedimetric measurements were performed using a PGSTAT302N (Metrohm Autolab, B.V., Utrecht, The Netherlands), controlled by the Nova 2.1 software, with a double-junction Ag/AgCl/3 M KCl/1 M LiOAc (Metrohm, Switzerland) as the reference and a platinum rod as the counter electrode. All potentiometric measurements were performed using a high-impedance input 16-channel EMF monitor (Lawson Laboratories, Inc., Malvern, PA) to record the signal with a double-junction Ag/AgCl/3 M KCl/1 M LiOAc (Metrohm, Switzerland) as the reference electrode. All electrochemical measurements were carried out in a Faraday cage to prevent unwanted noise. Pictures were taken using a Canon EOS 5D Mark III camera equipped with a Canon MP-E 65 mm f/2.8 macro lens. If needed, the pictures were processed with ImageJ. H NMR measurements were performed in deuterated chloroform at 400 MHz and FT-IR spectra were taken with a Tensor 27 IR instrument, both from Bruker. Differential scanning calorimetry (DSC) measurements were performed with a DSC 1 STARe system from Mettler Toledo under N_2_ from −90 to 180 °C with a 10 °C/min heating ramp.

### Electrode Preparation

A transducing layer of single-walled octadecylamine-functionalized carbon nanotubes was drop-cast onto the electrode body by preparing a 1 mg/mL solution in THF. A total amount of 6 μL (3 times 2 μL) of transducing cocktail was drop-cast onto the electrode body and left to air-dry for 30 min. For the case of cations (K^+^, H^+^, Ca^2+^), a membrane cocktail was prepared by dissolving 50 mg of self-plasticized PVC into 50 μL of THF (Selectophore grade). 10 mmol/kg NaTFPB was then dissolved in the cocktail along with 30 mmol/kg ionophore (Valinomycin for potassium, H-ionophore (I) for pH, and Calcium ionophore (IV) for calcium, respectively). For anions (NO_3_^–^), 10 mmol/kg of tridodecylmethylammonium nitrate (TDMAN) was dissolved along with 50 mg of self-plasticized PVC into 50 μL of THF. A total amount of 15 μL (3 times 5 μL) of membrane cocktail was drop-cast onto the electrode body and left to dry overnight. Prior to analysis, the sensors were conditioned in 0.1 M of the corresponding ion. For pH measurements, the electrodes were conditioned in a universal buffer solution (citric acid, boric acid, and phosphoric acid at 0.04 M) at pH 7. All further pH measurements were always carried out in the same universal buffer adjusted with 1 M NaOH. Selectivity coefficients were determined by the fixed interference method^57^ using solutions that were 1 M in chloride and 0.01 M in the hydroxide of the respective interfering cation. The pH of the solutions was adjusted by the addition of hydrochloric acid. All activity coefficients^53^ were calculated according to ref 53.

### Safety Warning on Organic Azides

Organic azides, much like sodium azide, pose significant safety risks because of their potential for violent decomposition. This instability is especially prevalent in low-molecular-weight organic azides where the N:C ratio is less than 3. Although specific substances such as PVC-azide **2**, 3-azidopropanol **6a**, 6-azidohexanol **6b**, and 10-azidodecanol **6c** have been observed to remain stable without signs of breakdown, rigorous safety protocols must remain a priority. It is important to exercise extreme care whenever synthesizing or handling any organic azide.^54^

#### Synthesis of PVC-Azide (**2**)

Synthesis of PVC-azide (PVC-N_3_) **2** was performed according to ref 51 (see Scheme 1). To a round-bottom flask filled with 20 mL of dimethylformamide, 2.0 g of poly­(vinyl chloride) **1** was allowed to completely dissolve at 65 °C followed by the addition of 2.1 g of sodium azide to the reaction flask. The reaction mixture was then stirred for 4 h. The polymer was then precipitated in 100 mL of ice-cold methanol. The resulting precipitate was then filtered through a Büchner funnel using a 0.45 μm nylon filter. The obtained polymer was then completely dissolved in 10 mL of dimethylformamide, precipitated in 100 mL of ice-cold methanol and filtered through a Büchner funnel using a 0.45 μm nylon filter. This process was repeated one more time, and the final resulting polymer was dried under vacuum for 3 days to give 1.82 g of a flocculent white solid. The azide modification was confirmed by FT-IR analysis (Figure S1). The degree of azide modification was estimated to be 27.1% by elemental analysis.

**Scheme 1:**
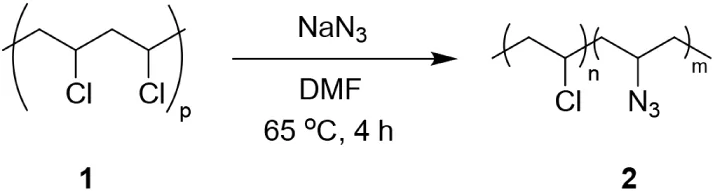
Synthesis of Modified PVC-Azide **2**

#### Synthesis of bis­(2-Ethylhexyl) Acetylenedicarboxylate (**5**)

bis­(2-Ethylhexyl) acetylenedicarboxylate **5** was synthesized according to ref 50 (Scheme 2). Acetylenedicarboxylic acid (4.05 g, 35.5 mmol), 2-ethyl-1-hexanol (10.3 g, 78.8 mmol), and para-toluenesulfonic acid monohydrate (0.45 g, 2.15 mmol) were dissolved in 56 mL of toluene in a round-bottom flask. The reaction mixture was refluxed for 1 h at 110 °C with a Dean–Stark trap to remove the water byproduct. The reaction mixture was then cooled down, and 40 mL of saturated sodium chloride solution was added. The aqueous layer was then extracted twice with 40 mL of hexane. The organic layers were combined, washed sequentially with 40 mL of water and saturated sodium bicarbonate, and finally dried over anhydrous magnesium sulfate. The volatiles were then evaporated under reduced pressure, and the resulting crude yellow oil was purified by column chromatography using 95:5 hexane/ethyl acetate as the eluent. 7.62 g of the final product was obtained as a colorless oil (61% yield). ^1^H NMR (Figure S2) (400 MHz, CDCl_3_): δ 4.21–4.09 (m, 4H), 1.70–1.54 (m, 2H), 1.39 (q, 12H), 1.35–1.20 (m, 3H), 0.89 (t, 12H).

**Scheme 2:**
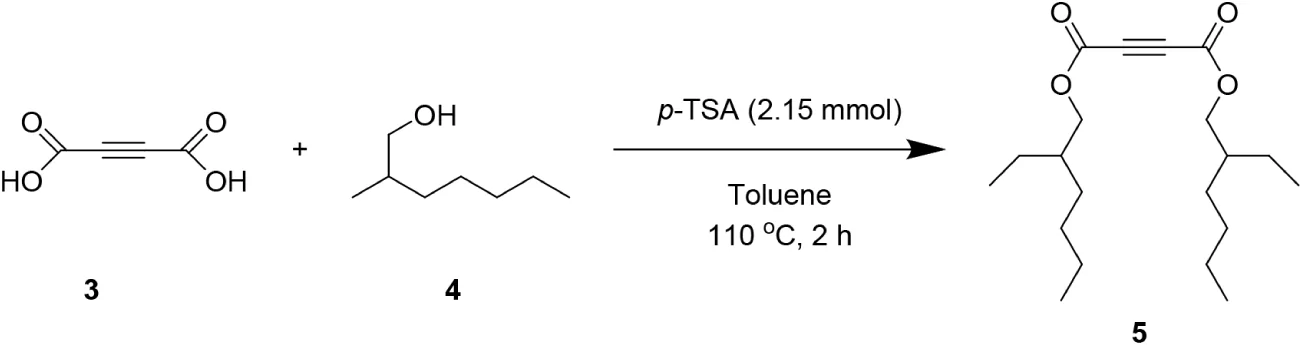
Synthesis of bis­(2-Ethylhexyl) Acetylenedicarboxylate **5**

#### Synthesis of bis­(2-Ethylhexyl)-1-(3-hydroxypropyl)-1H-1,2,3-triazole-4,5-dicarboxylate (**7a**)

The synthesis procedure of bis­(2-ethylhexyl)-1-(3-hydroxypropyl)-1H-1,2,3-triazole-4,5-dicarboxylate **7a** was adapted from ref 49 (Scheme 3). 3-Azido-1-propanol (0.5 g, 5.04 mmol) and bis­(2-ethylhexyl) acetylenedicarboxylate **5** (1.63 g, 4.62 mmol) were dissolved in 12 mL of chloroform. The reaction mixture was stirred at 50 °C for 24 h. The volatiles were then evaporated under reduced pressure, and the resulting crude colorless oil was purified by column chromatography using a 7:3 hexane/ethyl acetate as the eluent. 1.79 g of the final product was obtained as a colorless oil (88.0% yield). ^1^H NMR (Figure S3) (400 MHz, CDCl_3_): δ 4.73 (t, *J* = 6.7 Hz, 2H), 4.37–4.17 (m, 4H), 3.63 (t, 2H), 2.15 (tt, 2H), 1.69 (dh, 2H), 1.53–1.17 (m, 17H), 0.98–0.77 (m, 12H).

**Scheme 3:**
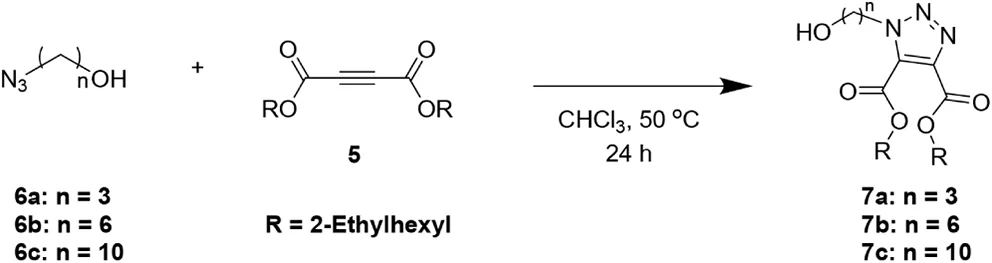
Synthesis of bis­(2-Ethylhexyl)-1-(n-hydroxyalkyl)-1H-1,2,3-triazole-4,5-dicarboxylate **7a**–**c**

#### Synthesis of bis­(2-Ethylhexyl)-1-(6-hydroxyhexyl)-1H-1,2,3-triazole-4,5-dicarboxylate (**7b**)

The synthesis procedure of bis­(2-ethylhexyl)-1-(6-hydroxyhexyl)-1H-1,2,3-triazole-4,5-dicarboxylate **7b** was similar to **7a** by using **6b** as the starting material (Scheme 3). 1.68 g of the final product was obtained as a colorless oil (83.0% yield). ^1^H NMR (Figure S4) (400 MHz, CDCl_3_): δ 4.58 (t, 2H), 4.35–4.20 (m, 4H), 3.62 (t, 2H), 1.91 (ddd, 2H), 1.80–1.61 (m, 2H), 1.55 (tt, 2H), 1.46–1.25 (m, 21H), 0.95–0.84 (m, 12H).

#### Synthesis of bis­(2-Ethylhexyl)-1-(10-hydroxydecyl)-1H-1,2,3-triazole-4,5-dicarboxylate (**7c**)

The synthesis procedure of bis­(2-ethylhexyl)-1-(10-hydroxydecyl)-1H-1,2,3-triazole-4,5-dicarboxylate **7c** was similar to **7a** by using **6c** as the starting material (Scheme 3). 1.17 g of the final product was obtained as a colorless oil (87.0% yield). ^1^H NMR (Figure S5) (400 MHz, CDCl_3_): δ 4.60–4.52 (m, 2H), 4.36–4.20 (m, 4H), 3.63 (t, 2H), 1.88 (p, 2H), 1.71 (dhept, 2H), 1.61–1.50 (m, 2H), 1.48–1.26 (m, 29H), 0.96–0.84 (m, 12H).

#### Synthesis of bis­(2-Ethylhexyl)-1-(3-(undec-10-ynoyloxy)­propyl)-1H-1,2,3-triazole-4,5-dicarboxylate (**9a**)

The synthesis procedure of bis­(2-ethylhexyl)-1-(3-(undec-10-ynoyloxy)­propyl)-1H-1,2,3-triazole-4,5-dicarboxylate **9a** was adapted from ref 51 (Scheme 4). bis­(2-Ethylhexyl)-1-(3-hydroxypropyl)-1H-1,2,3-triazole-4,5-dicarboxylate **7a** (1.62 g, 3.7 mmol), 10-undecynoic acid (0.5 g, 2.7 mmol), and para-toluenesulfonic acid monohydrate (28.0 mg, 0.15 mmol) were dissolved in 4 mL of toluene in a round-bottom flask. The mixture was stirred at reflux for 2 h using a Dean–Stark trap to remove the water byproduct. The volatiles were then evaporated under reduced pressure, and the resulting crude light brown oil was purified by column chromatography using 4:1 hexane/ethyl acetate as the eluent. 1.65 g of the final product was obtained as a colorless oil (67.5% yield). ^1^H NMR (Figure S6) (400 MHz, CDCl_3_): δ 4.70 (t, 2H), 4.35–4.20 (m, 4H), 4.14 (dt, 2H), 2.33–2.22 (m, 4H), 2.17 (td, 2H), 1.93 (t, 1H), 1.71 (dp, 2H), 1.63–1.56 (m, 2H), 1.48–1.22 (m, 26H), 0.98–0.84 (m, 12H).

**Scheme 4:**
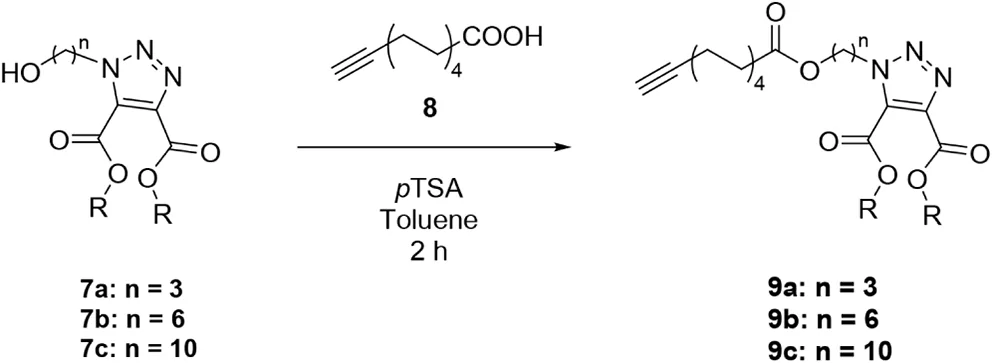
Synthesis of bis­(2-Ethylhexyl)-1-(n-(undec-10-ynoyloxy)­alkyl)-1H-1,2,3-triazole-4,5-dicarboxylate **9a**–**c**

#### Synthesis of bis­(2-Ethylhexyl)-1-(6-(undec-10-ynoyloxy)­hexyl)-1H-1,2,3-triazole-4,5-dicarboxylate (**9b**)

The synthesis procedure of bis­(2-ethylhexyl)-1-(6-(undec-10-ynoyloxy)­hexyl)-1H-1,2,3-triazole-4,5-dicarboxylate **9b** was similar to **9a** by using **7b** as the starting material (Scheme 4). 1.35 g of the final product was obtained as a colorless oil (67.0% yield). ^1^H NMR (Figure S7) (400 MHz, CDCl_3_): δ 4.57 (t, 2H), 4.27 (qd, 4H), 4.03 (t, 2H), 2.27 (t, 2H), 2.17 (td, 2H), 1.96–1.84 (m, 3H), 1.70 (dp, 2H), 1.64–1.56 (m, 4H), 1.44–1.22 (m, 29H), 0.95–0.84 (m, 12H).

#### Synthesis of bis­(2-Ethylhexyl)-1-(10-(undec-10-ynoyloxy)­propyl)-1H-1,2,3-triazole-4,5-dicarboxylate (**9c**)

The synthesis procedure of bis­(2-ethylhexyl)-1-(10-(undec-10-ynoyloxy)­decyl)-1H-1,2,3-triazole-4,5-dicarboxylate **9c** was similar to **9a** by using **7c** as the starting material (Scheme 4). 1.38 g of the final product was obtained as a colorless oil (69.0% yield). ^1^H NMR (Figure S8) (400 MHz, CDCl_3_): δ 4.56 (dd, 2H), 4.36–4.20 (m, 4H), 4.05 (t, 2H), 2.28 (t, 2H), 2.17 (td, 2H), 1.96–1.82 (m, 3H), 1.78–1.66 (m, 2H), 1.65–1.46 (m, 7H), 1.44–1.26 (m, 34H), 0.96–0.85 (m, 12H).

#### Synthesis of Self-Plasticized PVC **10a**

The synthesis procedure for self-plasticized PVC **10a** was adapted from ref 51 (Scheme 5). To a round-bottom flask filled with 5 mL of dimethylformamide (DMF), PVC-N_3_ **2** (120 mg) and bis­(2-ethylhexyl)-1-(3-(undec-10-ynoyloxy)­propyl)-1H-1,2,3-triazole-4,5-dicarboxylate **9a** (0.52 g, 0.86 mmol) were added and dissolved. To this reaction mixture, catalytic amounts of copper sulfate and l-ascorbic acid were added to promote the click reaction. The reaction mixture was heated and stirred at 45 °C for 3 days. The polymer was then dropwise precipitated in 100 mL of ice-cold methanol and obtained via filtration through a 0.45 μm nylon filter on a Büchner funnel. The obtained polymer was allowed to dissolve completely in 10 mL of tetrahydrofuran, precipitated in 100 mL of ice-cold methanol, and filtered through a 0.45 μm nylon filter on a Büchner funnel. The final product was dried under house vacuum for 3 days to ensure complete evaporation of the solvents, yielding 302.8 mg of green, elastic polymer. FT-IR spectra (Figure S9) confirmed the full conversion of the azide groups in PVC-N_3_. DSC (*T*_g_): −31.5 °C

**Scheme 5:**
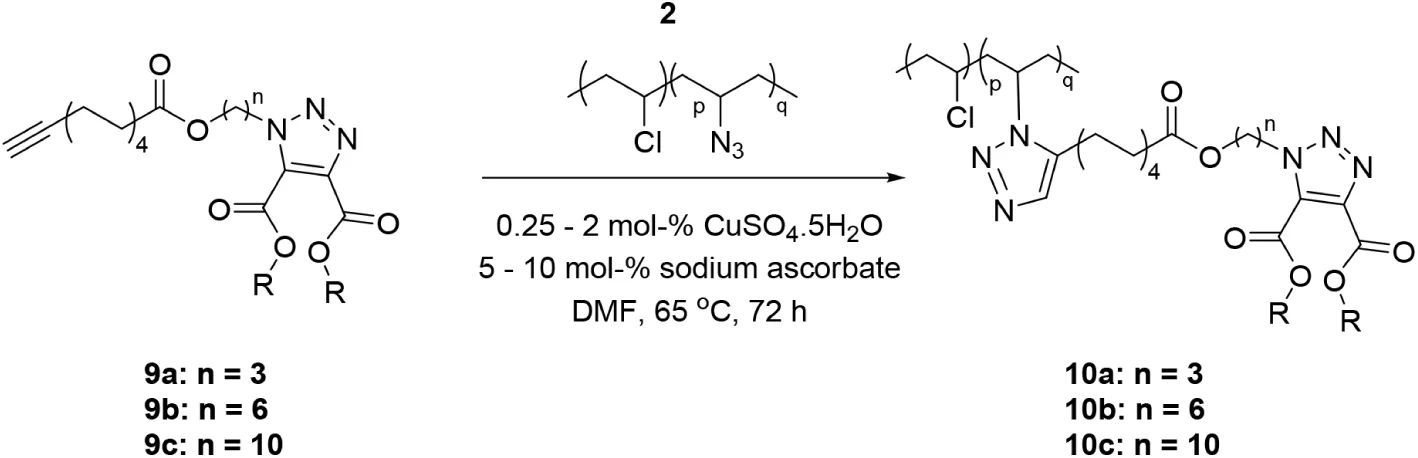
Synthesis of Self-Plasticized PVC **10a**–**c**

#### Synthesis of Self-Plasticized PVC **10b**

The synthesis procedure for self-plasticized PVC **10b** was identical to that of self-plasticized PVC **9b**. 150.6 mg of stretchy light-green polymer was isolated, and the conversion of the azide groups on the PVC backbone was estimated by FT-IR (Figure S10). DSC (*T*_g_): −22.82 °C

#### Synthesis of Self-Plasticized PVC **10c**

The synthesis procedure for self-plasticized PVC **10c** was identical to that of self-plasticized PVC **9c**. 130.8 mg of stretchy light-green polymer was isolated, and the conversion of the azide groups on the PVC backbone was estimated by FT-IR (Figure S11). DSC (*T*_g_): −22.94 °C

## Results and Discussion

The glass-transition temperature is an important physical property of a polymer that allows one to assess elasticity and malleability, important parameters of ion-selective membranes. Another parameter of high importance is the hydrophobicity of the polymer because it affects the response and overall stability of the membrane. Less hydrophobic (more hydrophilic) membranes are at a risk of forming a water layer at the interface of the transducing layer and membrane and may also exhibit poor dissolution of lipophilic membrane components, which is undesired.

Scheme 6 shows the overall synthesis of a range of self-plasticized polymers synthesized here that make use of long alkyl chains and one-pot click chemistry to graft all components onto the PVC-N_3_ to achieve the desired properties of a self-plasticized ion-selective membrane. The synthesis procedure is adapted from ref 51 and modified to obtain a polymer that is more hydrophobic and lipophilic by attaching linker compounds of various lengths between the plasticizer-like compound and the modified PVC-N_3_ polymer to achieve an optimal distance. bis­(2-Ethylhexyl) acetylenedicarboxylate (**5**) was synthesized via a Fischer esterification reaction between acetylenedicarboxylic acid and 2-ethyl-1-hexanol with *p*-toluenesulfonic acid as an acid catalyst, as detailed in Scheme 2. Taking the resulting ester **5**, a moiety **7a**–**c**, resembling the plasticizer bis­(2-ethylhexyl) phthalate (DEHP), was synthesized using thermal click chemistry^55^ between bis­(2-ethylhexyl) acetylenedicarboxylate (**2**) and three commercially available substrates, 3-azido-1-propanol (**6a**), 6-azido-1-hexanol (**6b**), and 10-azido-1-decanol (**6c**) via the 1,3-dipolar cycloaddition. Column chromatography purification ensured that no free alkyne was remaining for cross-reactivity in subsequent stages of synthesis.^56^

**Scheme 6:**
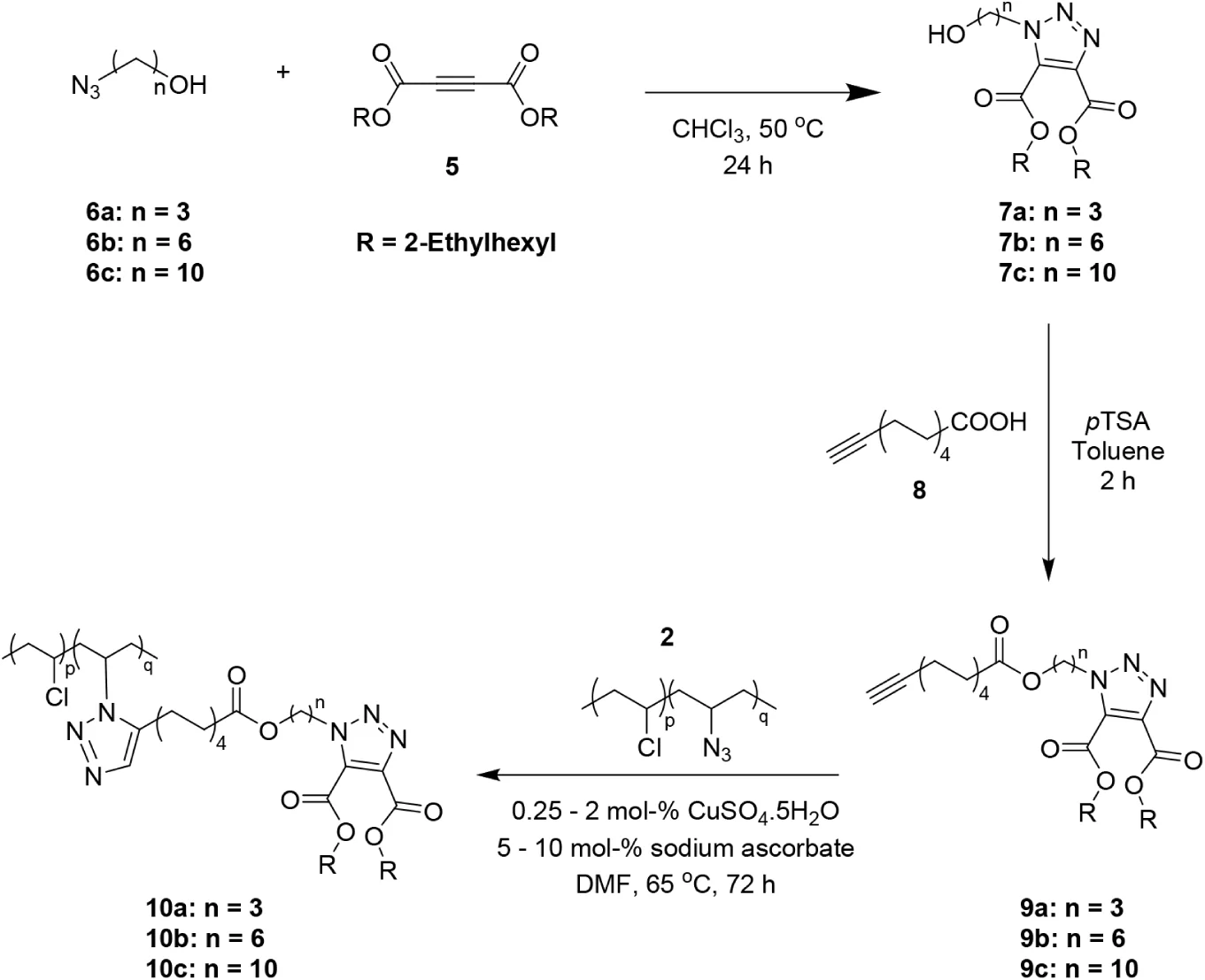
Overall Synthetic Scheme for Self-Plasticised PVC **10a–c**

A new, longer linker in the form of commercially available 10-undecynoic acid was also attached to the clicked product **7a**–**c** via a second Fischer esterification reaction to obtain **9a**–**c** and was purified similarly to **7a–c.** Finally, this ester **9a**–**c** was clicked to the previously synthesized PVC-N_3_ **2** via a copper-catalyzed click reaction that utilized copper sulfate along with ascorbic acid as the reducing agent to generate the necessary copper catalyst. The mixture was allowed to stir under low heat for 72 h to ensure complete reaction of the azide groups of PVC-N_3_ with the terminal alkyne of the ester **9a**–**c** which was confirmed via FT-IR (Figures S9–S11) After complete drying, the final polymer **10a**–**c** was obtained as a green, stretchy polymer that was chemically stable and synthetically reproducible by skipping the process of polymerization and only involving a covalent linkage between the plasticizer-like moiety and the existing modified polymer. The green color is attributed to the presence of residual copper trapped in the matrix of the polymer, which, despite multiple precipitations and washing with EDTA, proved to be persistent. This is likely due to it being trapped in the polymer layers, making it unfortunately too difficult to quantify. Hydrophobicity was tested by casting a drop of water on a solvent-cast polymer film and measuring the contact angle. A contact angle of 106.5° was obtained which indicates high hydrophobicity. The glass transition temperature of the synthesized polymers was measured using differential scanning calorimetry. They were in the range of −32 °C to −22 °C (see Experimental Section), which was higher than classical DEHP-plasticized PVC (−60 °C)^50^ but sufficient for successful casting of functional ion-selective membranes. The drop-casted self-plasticized membranes (Figure 1) displayed comparable elasticity and flexibility to classical plasticized PVC membranes when exposed to mechanical stress by pulling on the sides. The wrinkling and shriveling on the sides shown in Figure 1B are from the peeling of the drop-cast polymer from the glass slide.

**Figure 1:**
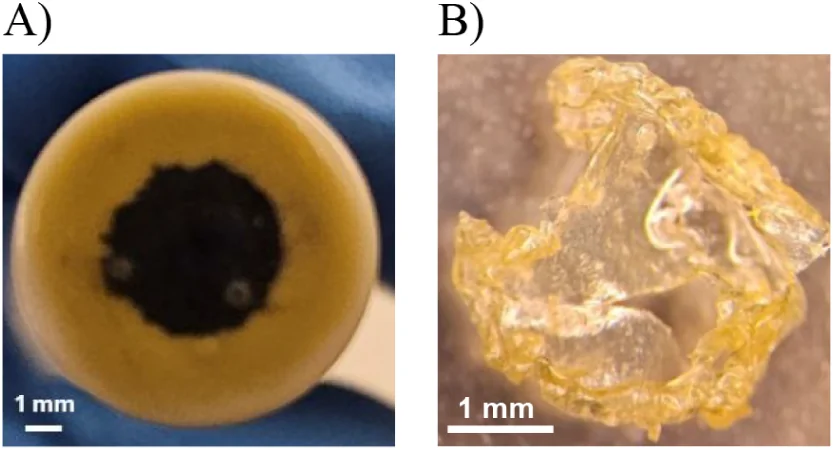
Pictures of ISE membranes prepared with self-plasticized (linker *n* = 3) 27% modified PVC. A) Ion-selective membrane prepared by drop-casting 1 × 50 μL of (*n* = 3) membrane cocktail. B) Ion-selective membrane (prepared by drop-casting on a glass slide) under a light microscope (40×).

In an initial evaluation of a model self-plasticized PVC polymer for further experimentation, potassium-selective membranes were prepared using the polymers **10a, 10b, and 10c** by dissolving them separately in THF along with the ion exchanger, NaTFPB, and the ionophore valinomycin. This membrane cocktail was drop-cast over a transducing layer of fCNTs. The electrodes were allowed to condition overnight in a 0.1 M KCl solution. A high resistance for all three membranes was observed (Figure S13C,E,F), likely because the plasticizer analogue was directly grafted onto the PVC background, thus reducing its mobility.

The membrane response to potassium ion activity was then evaluated, and a difference was observed depending on the azido-alcohol linker used to synthesize the plasticizer analogue (Figure 2). A practically useful linear response range was observed between log *a*_K^+^_ = −4.5 and −1 for the different linkers, which is slightly reduced compared to regular potassium ion-selective electrodes. The C_3_ linker (**10a**) and C_10_ linker (**10c**) exhibited much better slopes in comparison to the C_6_ linker (**10b**). An increased linker chain length should result in higher elasticity and hydrophobicity and give better performance, so the observed irregular trend is surprising with no explanation in the reviewed literature. One reason may be the physical structure of the polymer itself which might have changed during the synthesis (potential cross-linking), owing to which a less-than-optimum performance was observed, even though the glass transition temperatures of the linkers were comparable (C_3_ linker = −31.5 °C, C_6_ linker = −22.82 °C, C_10_ linker = −22.94 °C) and relatively close to each other. Additionally, the six times higher resistance values in the case of C_6_ linker and three times higher values for C_10_ linker could be the reason behind the reduced performance in comparison to the C_3_ linker. An in-depth study on the influence of linker length on membrane response is considered for the future to gain a better understanding.

**Figure 2:**
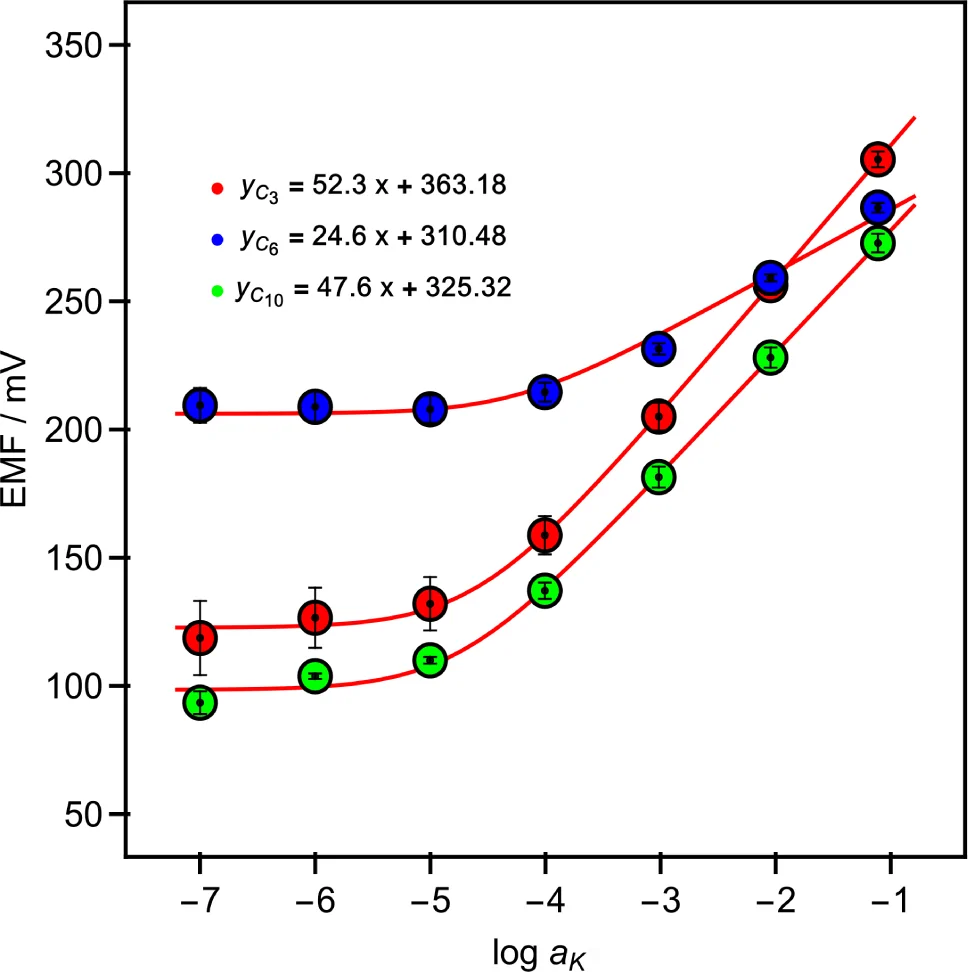
Potentiometric response of other K^+^-selective self-plasticized 27% modified PVCs **10a**–**c**. Blue circles represent C_3_ (*n* = 3) **10a,** red circles represent C_6_ (*n* = 6) **10b** linker PVC, and green circles represent C_10_ (*n* = 10) linker **10c**. Error bars are standard deviations (*n* = 3).

Based on the above results, C_3_ linker **10a** was chosen as a model substrate to create ion-selective membranes with a range of compositions to make them responsive to different clinically relevant ions (see Experimental Section for details). The response to calcium ions (Figure S12) was tested over multiple days after conditioning overnight in 0.1 M CaCl_2_ solution and displayed a linear response between log *a*_*Ca*_ = −5.5 and −1 (Figure 3). The total *E* ^0^ value change from day 1 to day 5 was just 0.3 mV, suggesting high stability of this membrane material. Impedimetric measurements gave a resistance of 13.86 MΩ (Figure S13A), which is higher than the classical membranes with otherwise the same composition.

**Figure 3:**
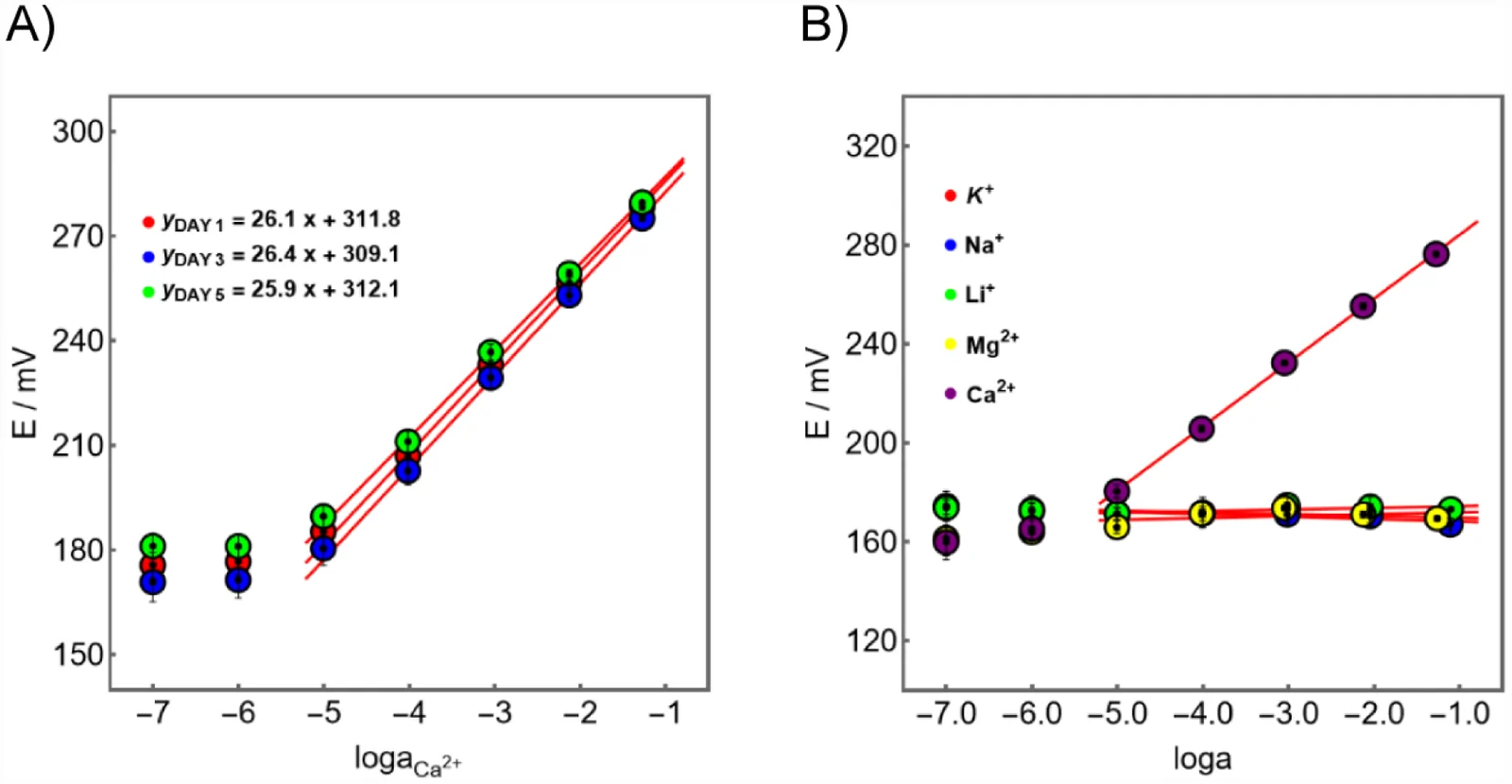
Potentiometric response of A) Ca^2+^-selective self-plasticized (**10a**) 27% modified PVC. Response after initial conditioning (red circles), after 3 days (blue circles), and after 5 days (green circles). B) EMF response of Ca^2+^-selective membrane in the presence of interfering ions (Red—K^+^, Blue—Na^+^, Green—Li^+^, Yellow—Mg^2+^, Violet—Ca^2+^) using MSSM. Error bars are standard deviations (*n* = 3).

In addition, unbiased selectivity coefficients were determined using the modified separate solution method (MSSM)^57^ where the response toward the interfering ion is measured before exposure to the primary ion of interest at varying concentrations. In MSSM, the electrodes are calibrated with interfering ions first before doing the same with the primary ion. They are conditioned in a 1 mM solution of the corresponding ion for 10–15 min before any calibration. The experimental sequence used here involved conditioning in a 1 mM MgCl_2_ solution followed by calibrations in chloride solutions of Li^+^, Na^+^, K^+^, Mg^2+^, and lastly the primary ion Ca^2+^ from the range of 10^–7^ M to 10^–1^ M. The Nicolsky–Eisenman equation was used to estimate the selectivity coefficient:

1 $$\log K_{ij}^{\mathrm{Pot}}=\frac{\left(E_{j}-E_{i}\right)z_{i}F}{2.303RT}+\log\frac{a_{i}}{a_{j}^{z_{i}/z_{j}}}$$

where $\log K_{ij}^{\mathrm{Pot}}$ is the selectivity coefficient of the primary ion (*i*) over the interfering ion (*j*), *E_j_* is the measured potential value of the interfering ion, *E_i_* is the measured potential value of the primary ion, *z_i_* is the charge of the primary ion, *z_j_* is the charge of the interfering ion, *a_j_* is the activity of the interfering ion, and *a_i_* is the activity of the primary ion. All calculated selectivity coefficients for the self-plasticized calcium-selective membrane are given in Table 1.

**Table 1:** Calculated Potentiometric Selectivity Coefficients for Calcium-Selective Electrode Prepared with Self-Plasticized PVC **10a** by MSSM

J	$\log K_{{\mathrm{Ca}}^{2+},J}^{\mathrm{Pot}}\mathrm{over}\;J$
K^+^	–4.75 ± 0.08
Na^+^	–4.71 ± 0.07
Li^+^	–4.62 ± 0.02
Mg^2+^	–4.63 ± 0.07

The selectivity coefficient over magnesium, $\log K_{{\mathrm{Ca}}^{2+},{\mathrm{Mg}}^{2+}}^{\mathrm{Pot}}$ = −4.63 ± 0.07, is excellent and in accordance with the literature value of ∼−4.0,^58^ as magnesium is a potentially competing ion in natural and biological samples. Nearly 5 orders of magnitude selectivity over magnesium is attractive and should ensure accurate detection of calcium in practical samples. Selectivity coefficients for other ions are slightly lower than the traditionally reported logarithmic selectivity coefficient values but are still adequate for most applications of practical relevance.

The applicability of the new self-plasticized matrix was also tested for the detection of pH with tridodecylamine as a hydrogen ion carrier (see Experimental Section for details). The potentiometric response of the resulting pH electrodes was tested for a period of 3 weeks.

As shown in Figure 4, the response slope of the pH electrode showed acceptable values over the course of 21 days. However, a shift in *E* ^0^ values of approximately −100 mV was observed from day 1 to day 5, which was reduced to about 30 mV by day 21. More surprisingly, a shift in the linear response range was observed from day 1 (pH 6 to 12) to day 5 (pH 4 to 10). This points to an apparent change in the p*K*_a_ value of the ionophore in the membrane, which may indicate reactivity of the tertiary amine group or more likely a chemical change in the polymer. A possible explanation for the *E* ^0^ drift could be the instability in adhesion of the polymer membrane to the electrode surface due to the formation of a water layer. A flattening response at higher H^+^ concentrations is normally explained by increased anion interferences for neutral-carrier electrodes.^59,60^ This is the only membrane exhibiting such unusual behavior, and additional experiments are planned in the future to expand the scope and develop a better understanding of the polymer characteristics and performance. The resistance of this membrane was estimated by impedimetric measurements and was observed to be of the same order as the calcium sensor (R = 3.24 MΩ, Figure S13B).

**Figure 4:**
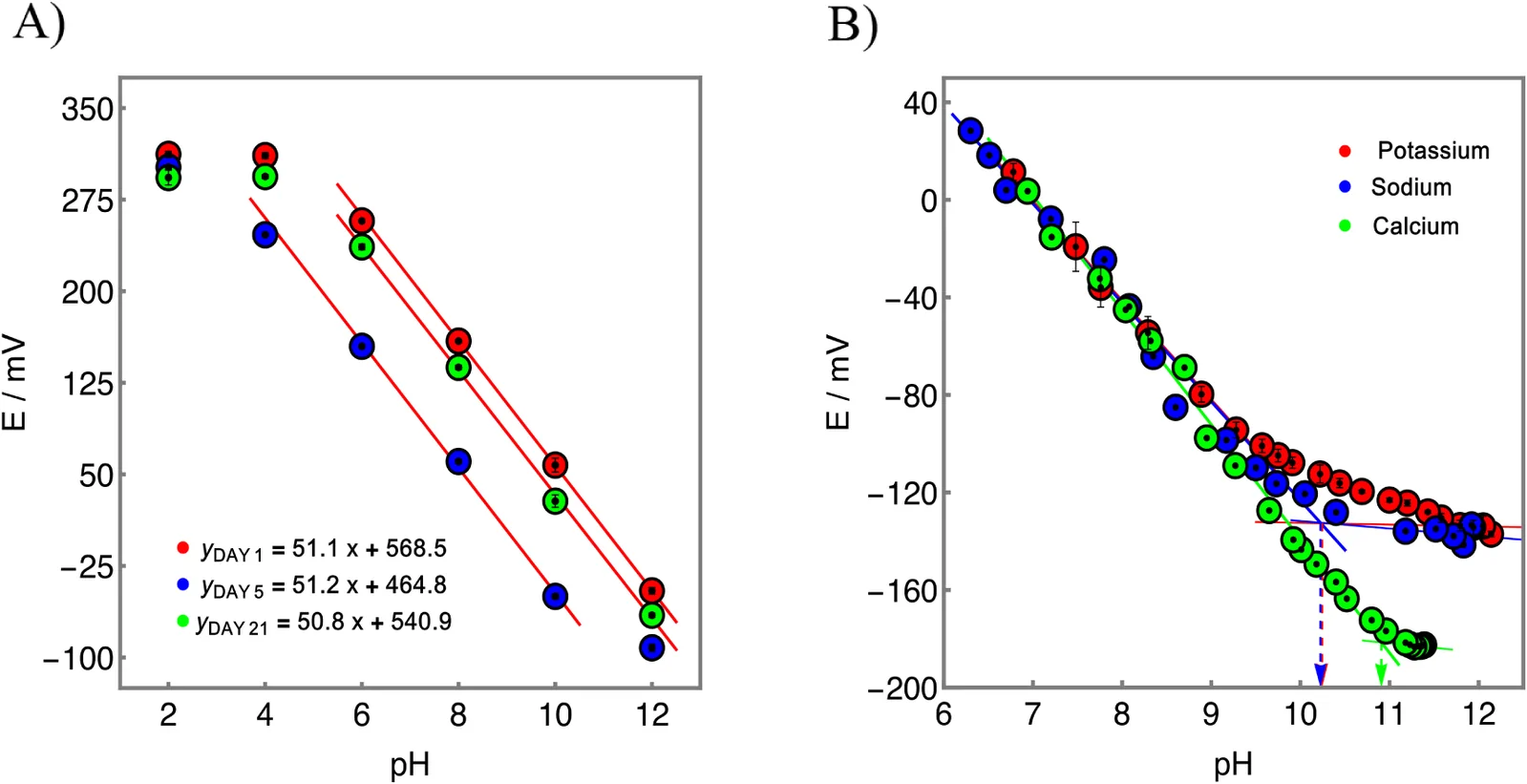
Potentiometric response of A) pH-selective self-plasticized (**10a)** 27% modified PVC after initial conditioning (red circles), after 5 days (blue circles), and after 21 days (green circles). Error bars are standard deviations (*n* = 3). B) EMF response of the pH sensor in the presence of interfering ions (Red—Potassium, Blue—Sodium, Green—Calcium) using FIM. The arrow indicates the lower detection limit. Error bars are standard deviations (*n* = 3).

The membranes prepared using the self-plasticized polymer **10a** also exhibited enhanced adhesion to the PEEK body located around the active area of the electrode. This characteristic is especially attractive for field applications, more precisely water analysis, where turbulent flow may promote membrane detachment and thus a premature deterioration of the sensor.

Measurements of unbiased selectivity coefficients were performed using the fixed interference method (FIM) in which the response to the hydrogen ion was recorded in a fixed background of an interfering ion. The intersection of the Nernstian response region (where the electrode responds to the primary ion) and the plateau region (where the curve flattens and the electrode responds to the interfering ion) gives the selectivity coefficient (Figure 3). It is also calculated using the following equation:

2 $$a_{i}\left(LDL\right)=K_{i,j}^{\mathrm{Pot}}a_{j}^{z_{i}/z_{j}}$$

where *a*_*i*_(*LDL*) is the activity of the primary ion at the detection limit, *a*_*j*_ the activity of the interfering ion, and *z*_*I*_ and *z*_*j*_ the valencies of the two ions. The determined selectivity coefficients for the self-plasticized pH sensor are given in Table 2, while Figure 3 shows the detection limit by the intersection of the Nernstian response range and plateau range.

**Table 2:** Calculated Potentiometric Selectivity Coefficients for pH Sensor Prepared with Self-Plasticized PVC **10a** by FIM

J	$\log K_{H^{+},J}^{\mathrm{Pot}}\mathrm{over}\;J$
K^+^	–10.2 ± 0.2
Na^+^	–10.3 ± 0.2
Ca^2+^	–10.9 ± 0.1

The selectivity coefficients over potassium, $\log K_{H^{+},K^{+}}^{\mathrm{Pot}}$ = −10.2, and sodium, $\log{\;K}_{H^{+},{\mathrm{Na}}^{+}}^{\mathrm{Pot}}$ = −10.3, are promising and deviate only slightly from the logarithmic selectivity coefficient of −10.4 for potassium^59^ and −9.8 for sodium reported in the literature.^59^ The selectivity coefficient over calcium was estimated as $\log{\;K}_{H^{+},{{\mathrm{Ca}}^{2}}^{+}}^{\mathrm{Pot}}$ = −10.9, which is nearly identical to the literature value of −11.1.^59^ A 10 orders of magnitude selectivity over potassium and sodium holds promising potential for real-world sample analysis.

In addition, the response for potassium in the polymer (**10a)** was also tested over multiple days and showed a higher linear response range from log *a*_*K*_ = −4.5 to −1.0 (Figure 5) with acceptable slope values in the initial days but appeared to deteriorate over the course of 5 days. The linear response range is slightly lower than that of regular potassium ion-selective electrodes. The *E* ^0^ values also differed and decreased from day 1 to day 5 which might potentially be attributed to the characteristics of the modified polymer. Impedimetric measurements were carried out and the resistance was estimated as 31.34 MΩ (Figure S13C) which was slightly higher than the values observed for the other ions.

**Figure 5:**
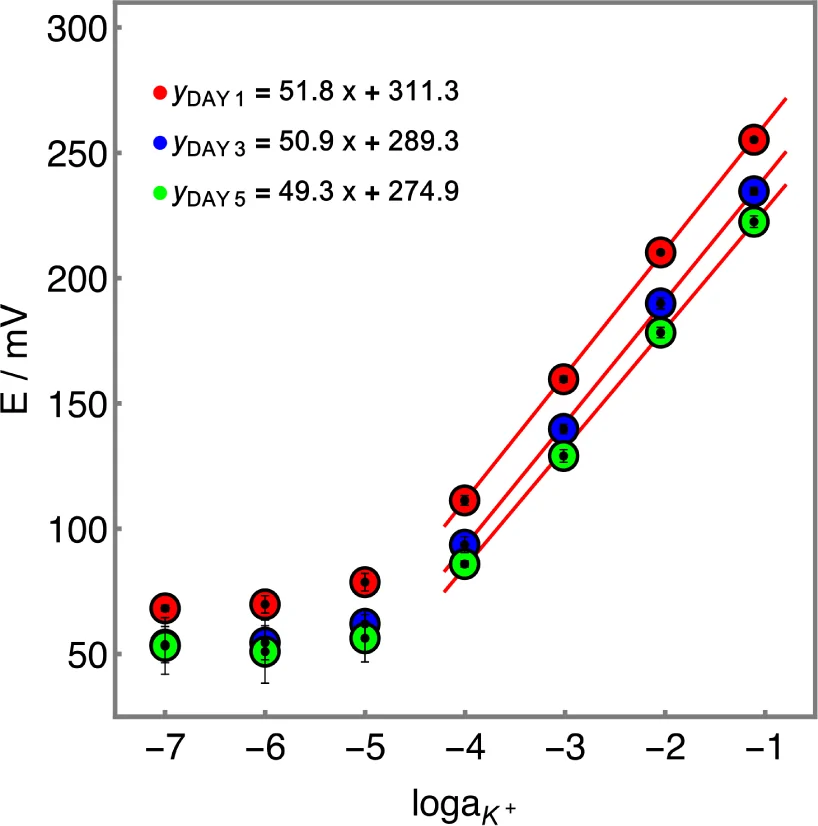
Potentiometric response of K^+^-selective self-plasticized (**10a)** 27% modified PVC. Response after initial conditioning (red circles), after 3 days (blue circles), and after 5 days (green circles). Error bars are standard deviations (*n* = 3).

Moreover, unbiased single-point selectivity coefficients were determined using the modified separate solution method (MSSM)^57^ where again the response toward the interfering ion was measured before the primary ion of interest at a fixed concentration and then estimated using the Nicolsky–Eisenman equation (eq 1). The calculated selectivity coefficients for the self-plasticized potassium-selective membrane are given in Table 3.

**Table 3:** Calculated Potentiometric Single-Point Selectivity Coefficients for Potassium-Selective Electrode Prepared with Self-Plasticized PVC **10a** by MSSM

J	$\log K_{K^{+},J}^{\mathrm{Pot}}\mathrm{over}\;J$
Na^+^	–2.67 ± 0.02
Ca^2+^	–3.22 ± 0.03
Mg^2+^	–3.12 ± 0.02

As shown in Table 3, the selectivity coefficient over sodium, $\log K_{K^{+},{\mathrm{Na}}^{+}}^{\mathrm{Pot}}$ = −2.67 ± 0.02 is lower than the reported literature value of −4.5 ^58,61^ for traditional membranes. The trend continues for selectivity coefficient values of calcium and potassium which despite the observed selectivity are found to be lower than the literature values of −7.5 for magnesium and −6.9 for calcium.

The response of the self-plasticized polymer **10a** was also tested for anions by using nitrate as the test ion. After preparation and conditioning overnight, a linear response was observed over a range of $\log a_{{\mathrm{NO}}_{3}^{-}}$ = −4.5 to −1, similar to the results for potassium (Figure 6). Similarly, the slope values decreased from day 1 to day 5 even though the *E* ^0^ values stabilized over the course of 5 days. The high resistance of the membrane was estimated to be R = 5.82 MΩ (Figure S13D), which was again similar to the other ion-sensing membranes based on this polymer matrix.

**Figure 6:**
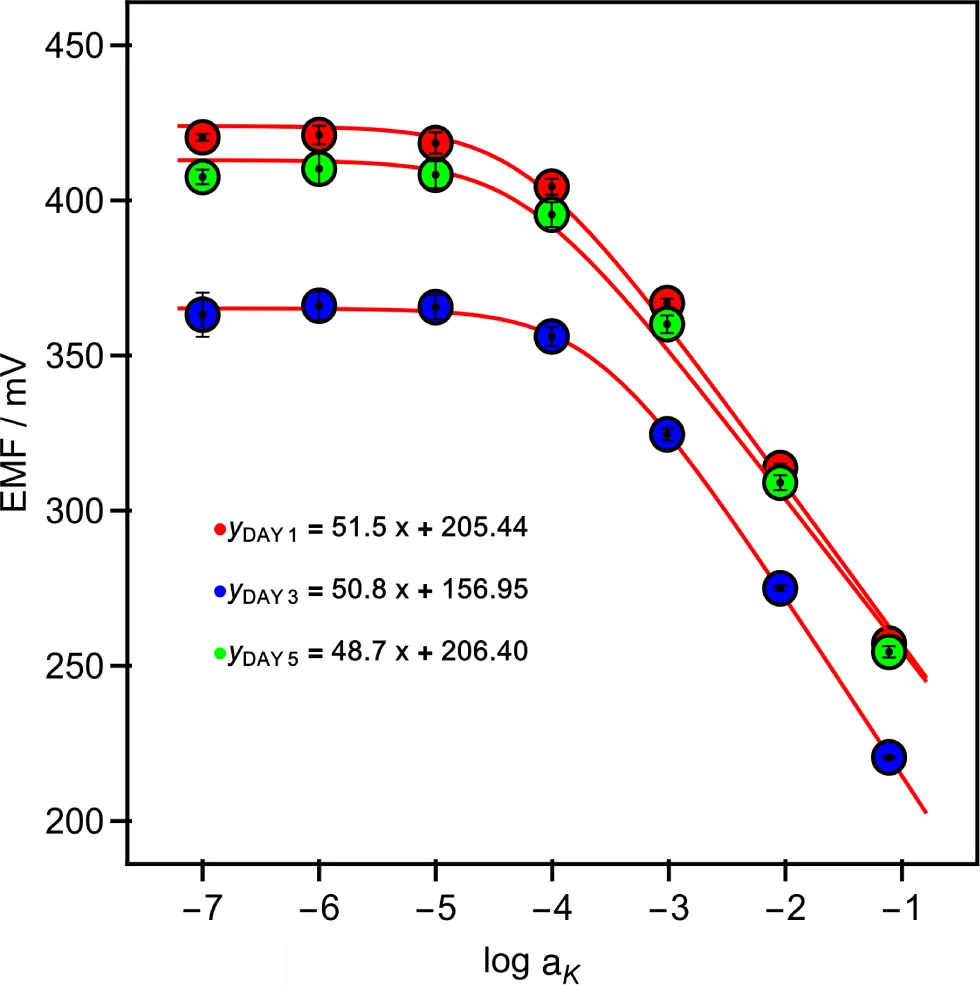
Potentiometric response of NO_3_^–^-selective self-plasticized (**10a**) 27% modified PVC. Response after initial conditioning (red circles), after 3 days (blue circles), and after 5 days (green circles). Error bars are standard deviations (*n* = 3).

Unbiased single-point selectivity coefficients were again determined using the modified separate solution method (MSSM).^57^ The calculated single-point selectivity coefficients for the self-plasticized nitrate-selective membrane are given in Table 4.

**Table 4:** Calculated Potentiometric Single-Point Selectivity Coefficients for Nitrate-Selective Electrode Prepared with Self-Plasticized PVC **10a** by MSSM

J	$\log K_{K^{+},J}^{\mathrm{Pot}}\mathrm{over}\;J$
Br^–^	–0.63 ± 0.02
Cl^–^	–2.18 ± 0.03
SO_4_^2–^	–2.85 ± 0.04

The selectivity coefficient over chloride, $\log K_{{\mathrm{NO}}_{3}^{-},{\mathrm{Cl}}^{-}}^{\mathrm{Pot}}$ = −2.18 ± 0.03, is observed to be slightly lower than the reported literature value of −2.9 for traditional membranes.^62^ This makes it usable for practical sample measurements.

To characterize plasticizer leaching from the membrane, a methanol test was conducted in which three classical PVC-DOS membranes and three self-plasticized PVC membranes were solvent-cast and exposed to methanol for 4 h to force the leakage of plasticizer from the membrane. Weight measurements were performed before and after the exposure to methanol.

A dramatic difference in the mass of the PVC/DOS membranes was observed before (7.63 mg ± 3.8%) and after (3.41 mg ± 4.1%) exposure to methanol. A second identical washing no longer reduced the observed mass, indicating the complete loss of plasticizer. In contrast, the self-plasticized PVC membrane showed no evidence of mass loss (changing from 0.95 mg ± 12% to 0.96 mg ± 11%), which confirmed that the plasticizer analog was strongly tethered onto the modified PVC backbone.

The methanol solutions after exposure were also analyzed via GC-MS (Figure S14), and a large, strong peak for the plasticizer DOS was visible in the case of plasticized PVC. No such peaks were observed in the case of the self-plasticized PVC **10a**, making the synthesized polymer an attractive option for cases where cytotoxicity was a potential concern.

## Conclusions

Through this work, we demonstrated the potential of self-plasticized PVC as a promising candidate as matrix for ion-selective electrodes. Multiple tethered plasticizer analogues were grafted onto azide-modified PVC using high-yielding, simple, and efficient one-pot click chemistry to give a flexible polymer without the additional need for a plasticizer. This efficient pathway can be used to synthesize new classes of self-plasticized polymers with varying chemical and physical properties. One example was used as a model system to show its applicability as a matrix for ion-selective electrodes for a library of ions. In particular, the polymer showed acceptable results in terms of the response and stability for the ions tested. There is evidence of a slow, long-term change in the solvent behavior of the polymers that merits further optimization, especially for the determination of pH. Leaching of polymer components is shown to be effectively eliminated, which is a major advantage compared with plasticized PVC.

## Supplementary Material



## Notes

FT-IR spectra of polymers, ^1^H NMR spectra of synthesized compounds, impedance spectra of all ion-selective polymer membranes, and GC-MS spectra of plasticizer leaching (PDF)

## ◆

PharmaBlock Sciences (Switzerland) AG, Bachtobelstrasse 5, 8810 Horgen, Switzerland
