# Supplementary material for: Leaching-Resistant Self-Plasticized Poly(Vinyl Chloride) (PVC) as a Matrix for Ion-Selective Electrodes
Source: ACS Meas Sci Au. 2026 Jul 26;6(5):1713–23. doi: 10.1021/acsmeasuresciau.6c00163 (PMC13693248; doi:10.1021/acsmeasuresciau.6c00163)
Supplement: Supplementary file 1 [file tg6c00163_si_001.pdf]

Supporting Information for:

Leaching-Resistant Self-Plasticised Poly(Vinyl Chloride) (PVC) as Matrix for Ion-Selective Electrodes

*Geetansh Gupta, Tara Forrest, Eric Bakker**

Department of Inorganic and Analytical Chemistry, University of Geneva, Quai Ernest-Ansermet 30, CH-1211 Geneva, Switzerland

**Corresponding author: Eric.Bakker@unige.ch*

Table of contents

Table of contents 2

Supporting Figures 4

Figure S1: FT-IR spectrum of non-modified PVC (black trace) and modified PVC-N_3 2 (red trace). The presence of azide groups on the PVC backbone can be confirmed by the typical azide band present at ~2100 cm-1. 4

Figure S2: ^{1}H NMR spectrum of di(2-ethylhexyl) acetylenedicarboxylate 5 (400 MHz, CDCl3 δ 4.21 – 4.09 (m, 4H), 1.70 – 1.54 (m, 2H), 1.39 (q, 12H), 1.35 – 1.20 (m, 3H), 0.89 (t, 12H). 4

Figure S3 : ^{1}H NMR spectrum of bis(2-ethylhexyl)-1-(3-hydroxypropyl)-1H-1,2,3-triazole-4,5-dicarboxylate 7a (400 MHz, $CDCl_3$): δ 4.73 (t, J = 6.7 Hz, 2H), 4.37 – 4.17 (m, 4H), 3.63 (t, 2H), 2.15 (tt, 2H), 1.69 (dh, 2H), 1.53 – 1.17 (m, 17H), 0.98 – 0.77 (m, 12H). 5

Figure S4 : ^{1}H NMR spectrum of bis(2-ethylhexyl)-1-(6-hydroxyhexyl)-1H-1,2,3-triazole-4,5-dicarboxylate 7b (400 MHz, $CDCl_3$): δ 4.58 (t, 2H), 4.35 – 4.20 (m, 4H), 3.62 (t, 2H), 1.91 (ddd, 2H), 1.80 – 1.61 (m, 2H), 1.55 (tt, 2H), 1.46 – 1.25 (m, 21H), 0.95 – 0.84 (m, 12H). 5

Figure S5 : ^{1}H NMR spectrum of bis(2-ethylhexyl)-1-(10-hydroxydecyl)-1H-1,2,3-triazole-4,5-dicarboxylate 7c (400 MHz, $CDCl_3$): δ 4.60 – 4.52 (m, 2H), 4.36 – 4.20 (m, 4H), 3.63 (t, 2H), 1.88 (p, 2H), 1.71 (dhept, 2H), 1.61 – 1.50 (m, 2H), 1.48 – 1.26 (m, 29H), 0.96 – 0.84 (m, 12H). 6

Figure S6: ^{1}H NMR spectrum of bis(2-ethylhexyl)-1-(3-(undec-10-ynoyloxy)propyl)-1H-1,2,3-triazole-4,5-dicarboxylate 9a (400 MHz, $CDCl_3$): δ 4.70 (t, 2H), 4.35 – 4.20 (m, 4H), 4.14 (dt, 2H), 2.33 – 2.22 (m, 4H), 2.17 (td, 2H), 1.93 (t, 1H), 1.71 (dp, 2H), 1.63 – 1.56 (m, 2H), 1.48 – 1.22 (m, 26H), 0.98 – 0.84 (m, 12H). 6

Figure S7: ^{1}H NMR spectrum of bis(2-ethylhexyl)-1-(6-(undec-10-ynoyloxy)hexyl)-1H-1,2,3-triazole-4,5-dicarboxylate 9b (400 MHz, $CDCl_3$): δ 4.57 (t, 2H), 4.27 (qd, 4H), 4.03 (t, 2H), 2.27 (t, 2H), 2.17 (td, 2H), 1.96 – 1.84 (m, 3H), 1.70 (dp, 2H), 1.64 – 1.56 (m, 4H), 1.44 – 1.22 (m, 29H), 0.95 – 0.84 (m, 12H). 7

Figure S8: ^{1}H NMR spectrum of bis(2-ethylhexyl)-1-(10-(undec-10-ynoyloxy)decyl)-1H-1,2,3-triazole-4,5-dicarboxylate 9c (400 MHz, $CDCl_3$): δ 4.56 (dd, 2H), 4.36 – 4.20 (m, 4H), 4.05 (t, 2H), 2.28 (t, 2H), 2.17 (td, 2H), 1.96 – 1.82 (m, 3H), 1.78 – 1.66 (m, 2H), 1.65 – 1.46 (m, 7H), 1.44 – 1.26 (m, 34H), 0.96 – 0.85 (m, 12H). 8

Figure S9: FT-IR spectrum of non-modified PVC (black trace) and self-plasticized PVC polymer 10a (red trace). The final click reaction can be confirmed by the absence of azide groups on the PVC backbone, characterised by the disappearance of the typical azide band present at ~2100 cm^{-1}. 8

Figure S10: FT-IR spectrum of non-modified PVC (black trace) and self-plasticized PVC polymer 10b (red trace). The final click reaction can be confirmed by the absence of azide groups on the PVC backbone, characterised by the disappearance of the typical azide band present at ~2100 cm^{-1}. 9

Figure S11: FT-IR spectrum of non-modified PVC (black trace) and self-plasticized PVC polymer 10c (red trace). The final click reaction can be confirmed by the absence of azide groups on the PVC backbone, characterised by the disappearance of the typical azide band present at ~2100 cm^{-1}. 9

Figure S12: Real time potential response for calcium-selective membrane ranging from concentration 10^{-7} M to 10^{-1} M. 10

Figure S13: Impedance spectra for A) calcium-selective membrane in 0.1 M $CaCl_2$, B) pH electrode in universal buffer 0.04M pH 7, C) potassium-selective membrane in 0.1 M KCl, D) nitrate-selective electrode in universal buffer 0.1 M KNO_3, E) C_6 linker potassium-selective membrane in 0.1 M KCl, F) C_{10} linker potassium-selective membrane in 0.1 M KCl Reference electrode: double-junction Ag/AgCl/ 3 M KCl/ 1 M LiOAc and counter electrode: Pt rod. Impedance measurements taken at OCP with 100 mV amplitude at a frequency range of 1 MHz to 100 mHz. Resistance estimated using semi-circle fit. 12

Figure S14: GC-MS spectra of methanol solution of a classical PVC/DOS membrane when exposed to methanol for 4 h. Pink trace - self-plasticized PVC membrane, Blue trace – PVC/DOS membrane, black trace – standard sample of DOS. The tall peak at 21.25 min indicates the plasticizer (DOS) leaching out. 12

Supporting Figures

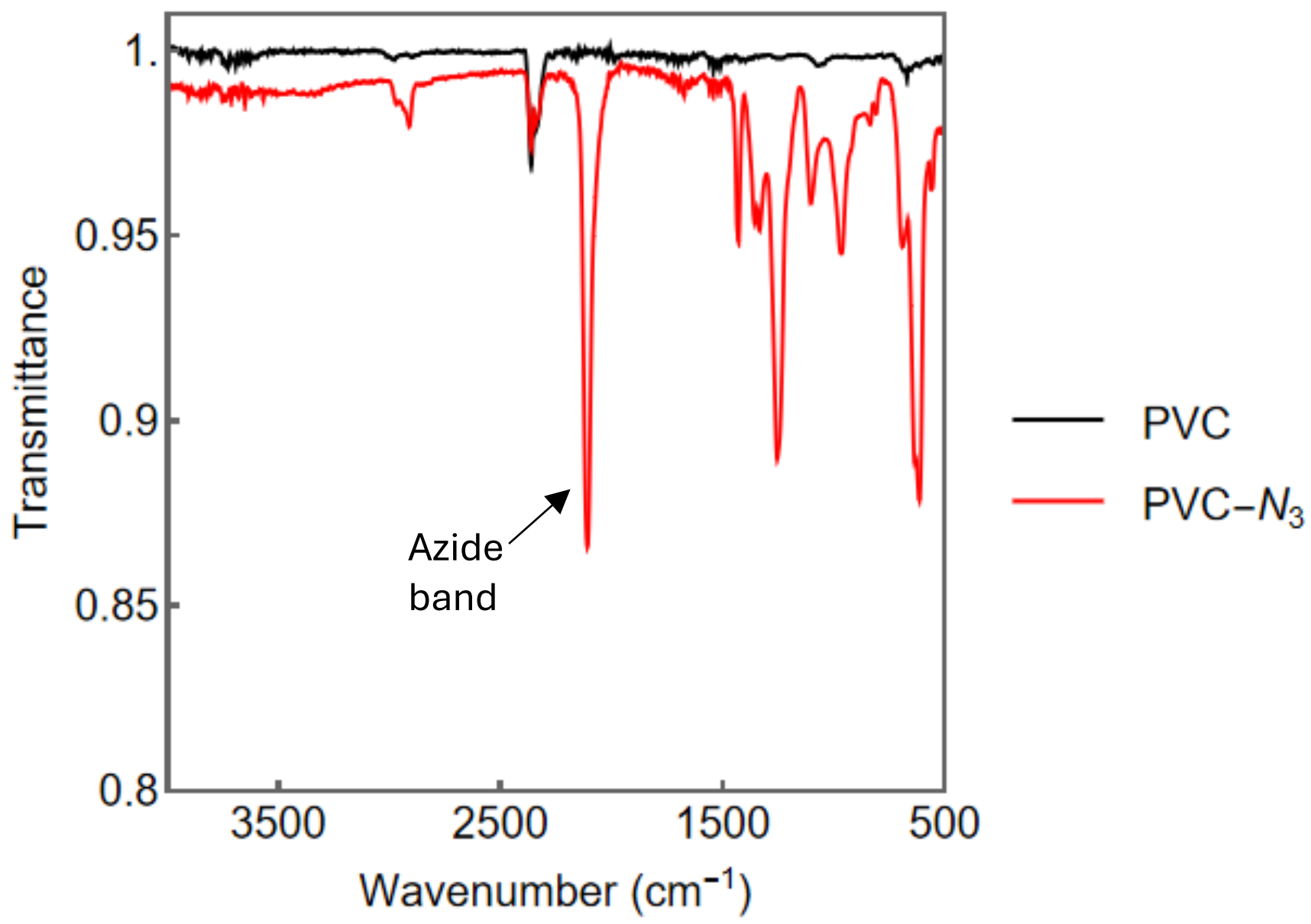

Figure S1: FT-IR spectrum of non-modified PVC (black trace) and modified PVC-N_3 2 (red trace). The presence of azide groups on the PVC backbone can be confirmed by the typical azide band present at ~2100 cm-1.

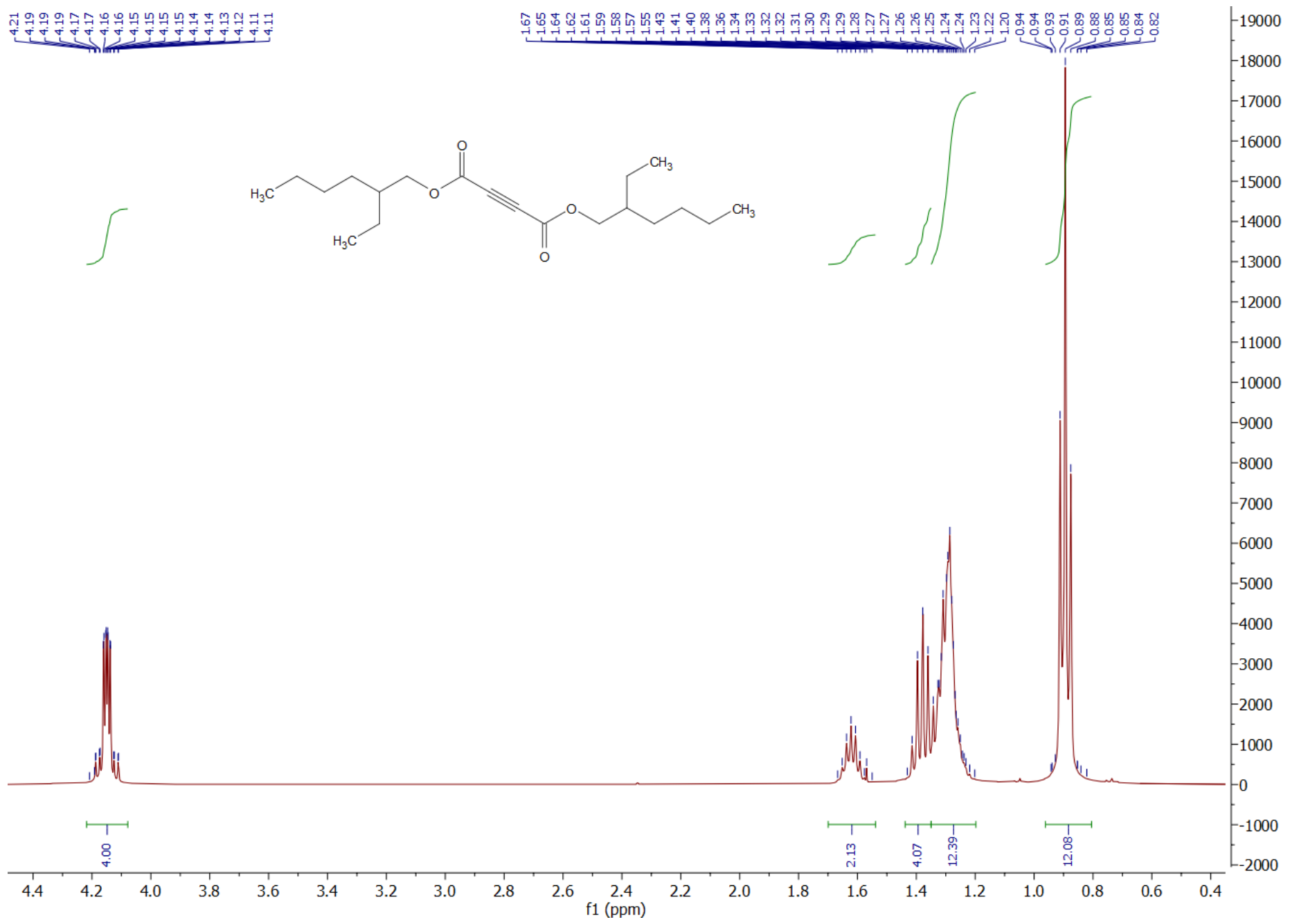

Figure S2: ^{1}H NMR spectrum of di(2-ethylhexyl) acetylenedicarboxylate 5 (400 MHz, $CDCl_3$ δ 4.21 – 4.09 (m, 4H), 1.70 – 1.54 (m, 2H), 1.39 (q, 12H), 1.35 – 1.20 (m, 3H), 0.89 (t, 12H).

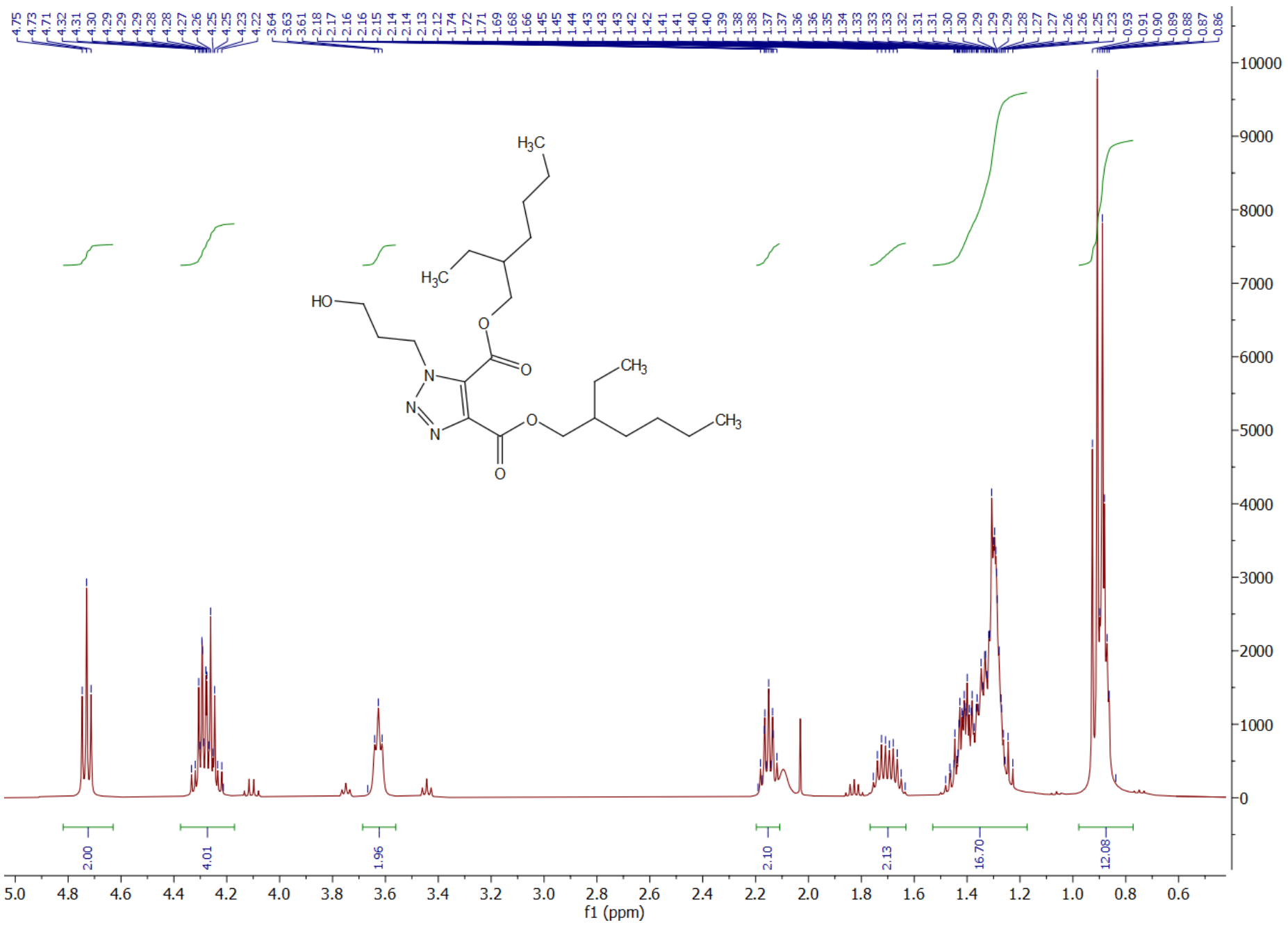

Figure S3 : ^{1}H NMR spectrum of bis(2-ethylhexyl)-1-(3-hydroxypropyl)-1H-1,2,3-triazole-4,5-dicarboxylate 7a (400 MHz, $CDCl_3$): δ 4.73 (t, *J* = 6.7 Hz, 2H), 4.37 – 4.17 (m, 4H), 3.63 (t, 2H), 2.15 (tt, 2H), 1.69 (dh, 2H), 1.53 – 1.17 (m, 17H), 0.98 – 0.77 (m, 12H).

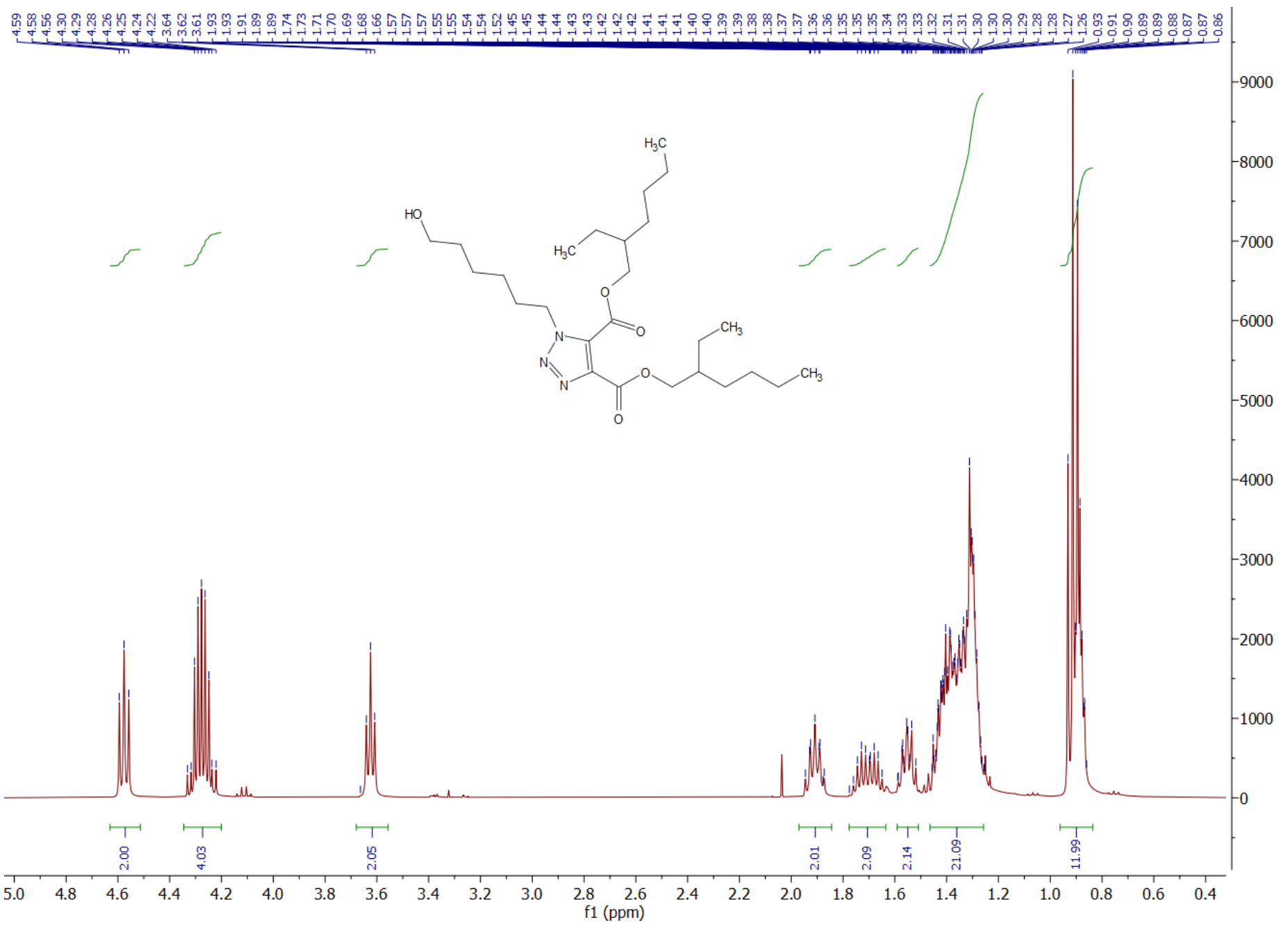

Figure S4 : ^{1}H NMR spectrum of bis(2-ethylhexyl)-1-(6-hydroxyhexyl)-1H-1,2,3-triazole-4,5-dicarboxylate 7b (400 MHz, $CDCl_3$): δ 4.58 (t, 2H), 4.35 – 4.20 (m, 4H), 3.62 (t, 2H), 1.91 (ddd, 2H), 1.80 – 1.61 (m, 2H), 1.55 (tt, 2H), 1.46 – 1.25 (m, 21H), 0.95 – 0.84 (m, 12H).

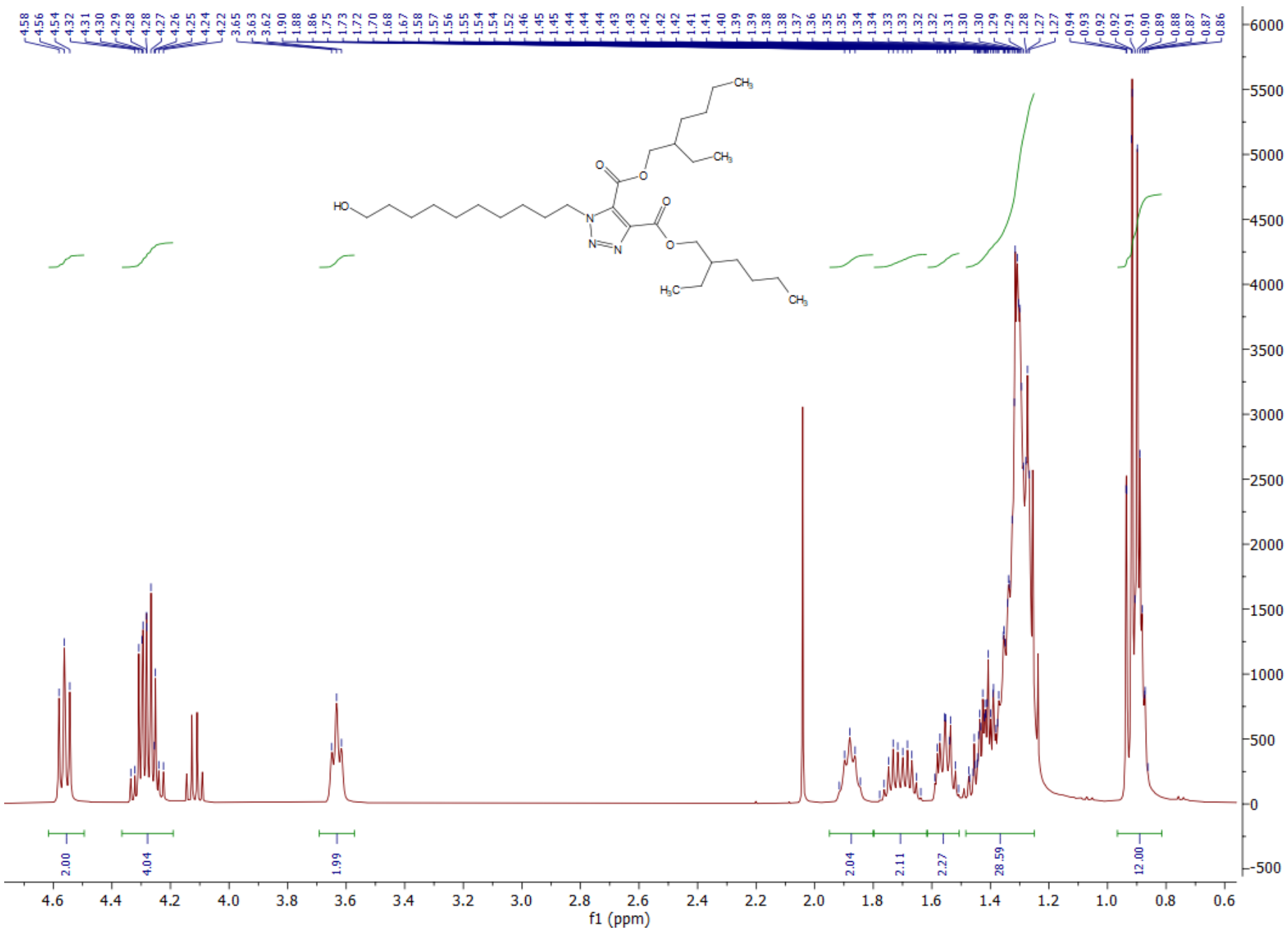

Figure S5 : ¹H NMR spectrum of bis(2-ethylhexyl)-1-(10-hydroxydecyl)-1H-1,2,3-triazole-4,5-dicarboxylate 7c (400 MHz, $CDCl_3$): δ 4.60 – 4.52 (m, 2H), 4.36 – 4.20 (m, 4H), 3.63 (t, 2H), 1.88 (p, 2H), 1.71 (dhept, 2H), 1.61 – 1.50 (m, 2H), 1.48 – 1.26 (m, 29H), 0.96 – 0.84 (m, 12H).

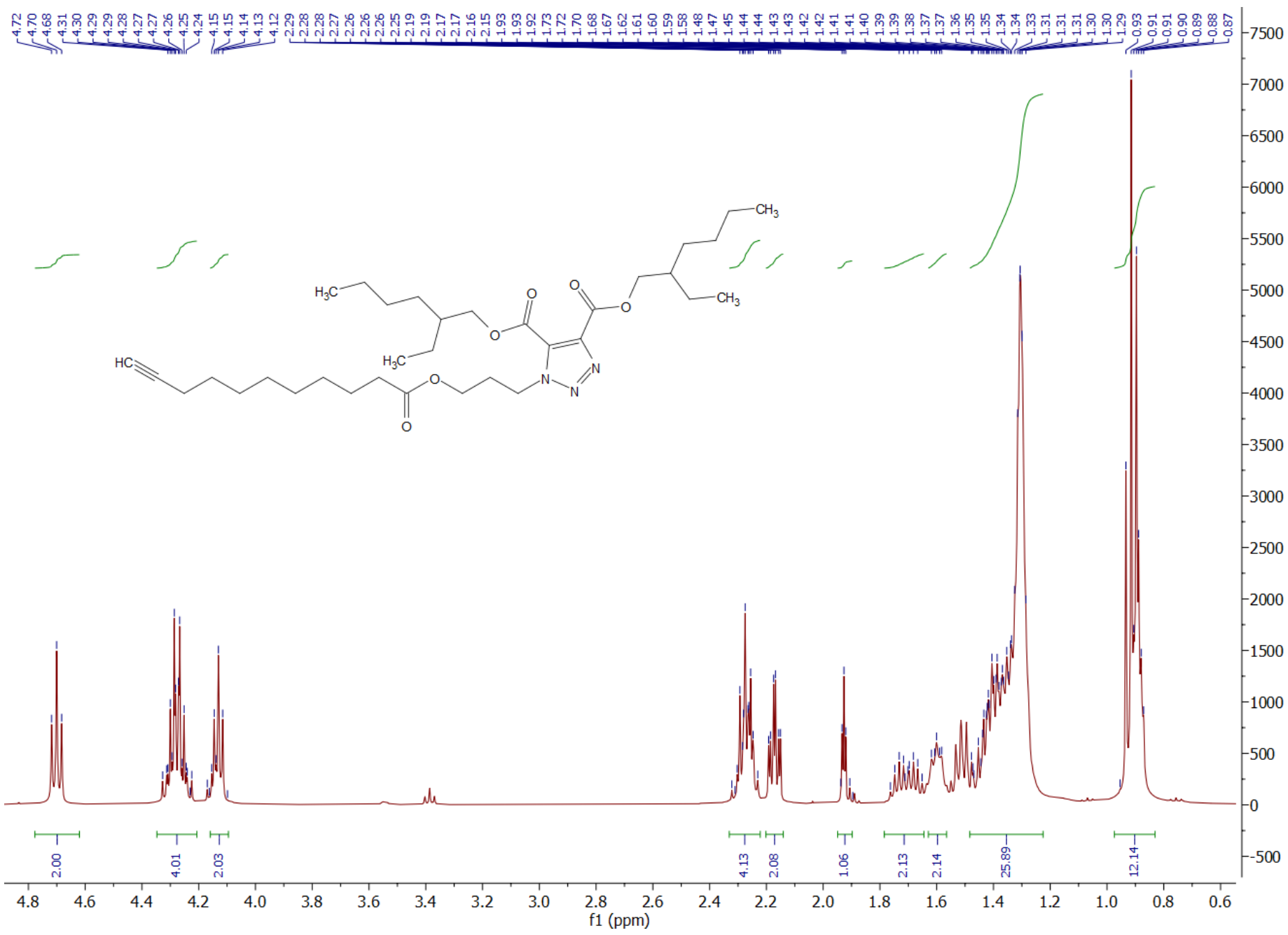

Figure S6: ¹H NMR spectrum of bis(2-ethylhexyl)-1-(3-(undec-10-ynoyloxy)propyl)-1H-1,2,3-triazole-4,5-dicarboxylate 9a (400 MHz, $CDCl_3$): δ 4.70 (t, 2H), 4.35 – 4.20 (m, 4H), 4.14 (dt, 2H), 2.33 – 2.22 (m, 4H), 2.17 (td, 2H), 1.93 (t, 1H), 1.71 (dp, 2H), 1.63 – 1.56 (m, 2H), 1.48 – 1.22 (m, 26H), 0.98 – 0.84 (m, 12H).

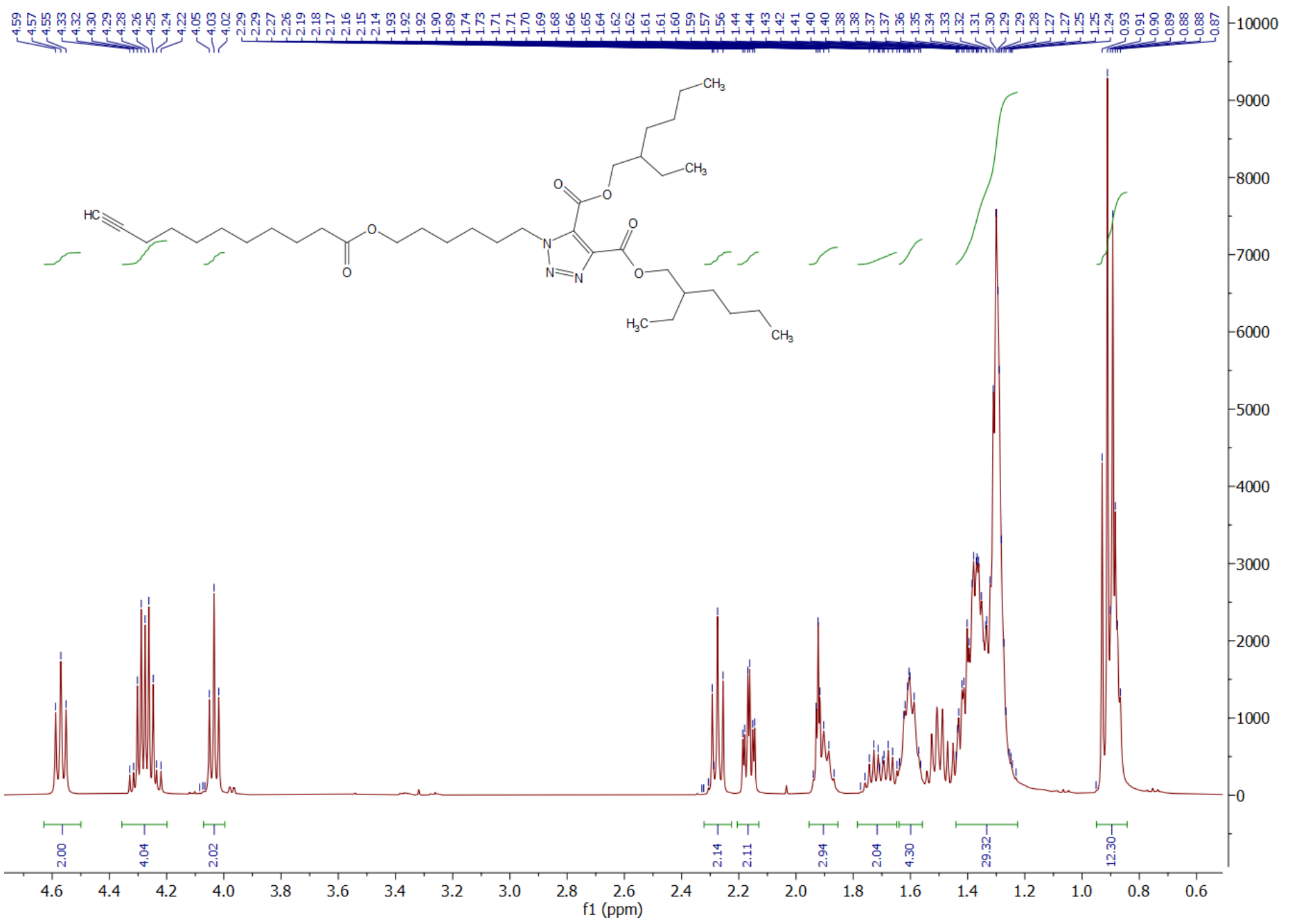

Figure S7: ^{1}H NMR spectrum of bis(2-ethylhexyl)-1-(6-(undec-10-ynoyloxy)hexyl)-1H-1,2,3-triazole-4,5-dicarboxylate 9b (400 MHz, $CDCl_3$): δ 4.57 (t, 2H), 4.27 (qd, 4H), 4.03 (t, 2H), 2.27 (t, 2H), 2.17 (td, 2H), 1.96 – 1.84 (m, 3H), 1.70 (dp, 2H), 1.64 – 1.56 (m, 4H), 1.44 – 1.22 (m, 29H), 0.95 – 0.84 (m, 12H).

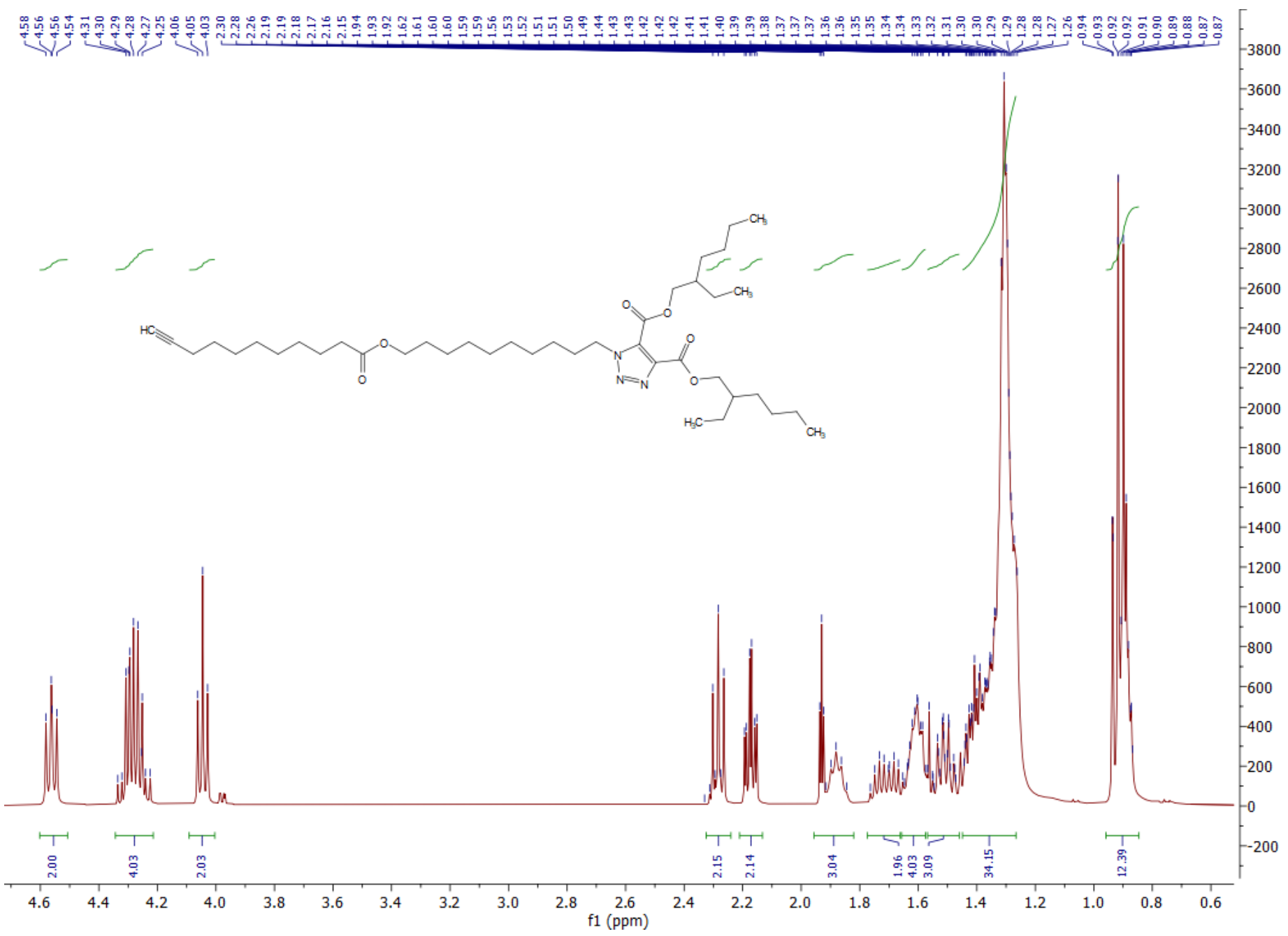

Figure S8: ^{1}H NMR spectrum of bis(2-ethylhexyl)-1-(10-(undec-10-ynoyloxy)decyl)-1H-1,2,3-triazole-4,5-dicarboxylate 9c (400 MHz, $CDCl_3$): δ 4.56 (dd, 2H), 4.36 – 4.20 (m, 4H), 4.05 (t, 2H), 2.28 (t, 2H), 2.17 (td, 2H), 1.96 – 1.82 (m, 3H), 1.78 – 1.66 (m, 2H), 1.65 – 1.46 (m, 7H), 1.44 – 1.26 (m, 34H), 0.96 – 0.85 (m, 12H).

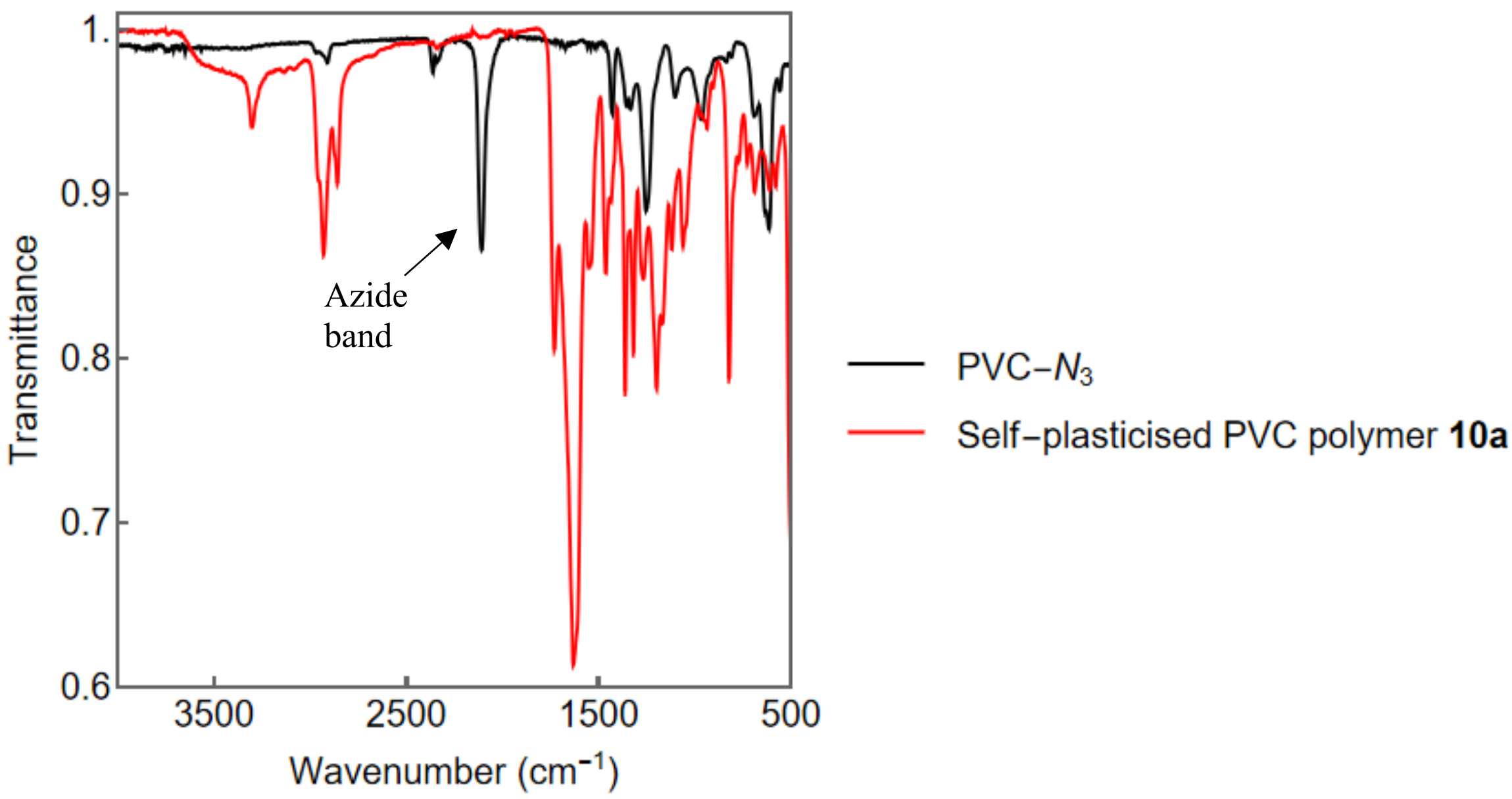

Figure S9: FT-IR spectrum of non-modified PVC (black trace) and self-plasticized PVC polymer 10a (red trace). The final click reaction can be confirmed by the absence of azide groups on the PVC backbone, characterised by the disappearance of the typical azide band present at ~2100 cm^{-1}.

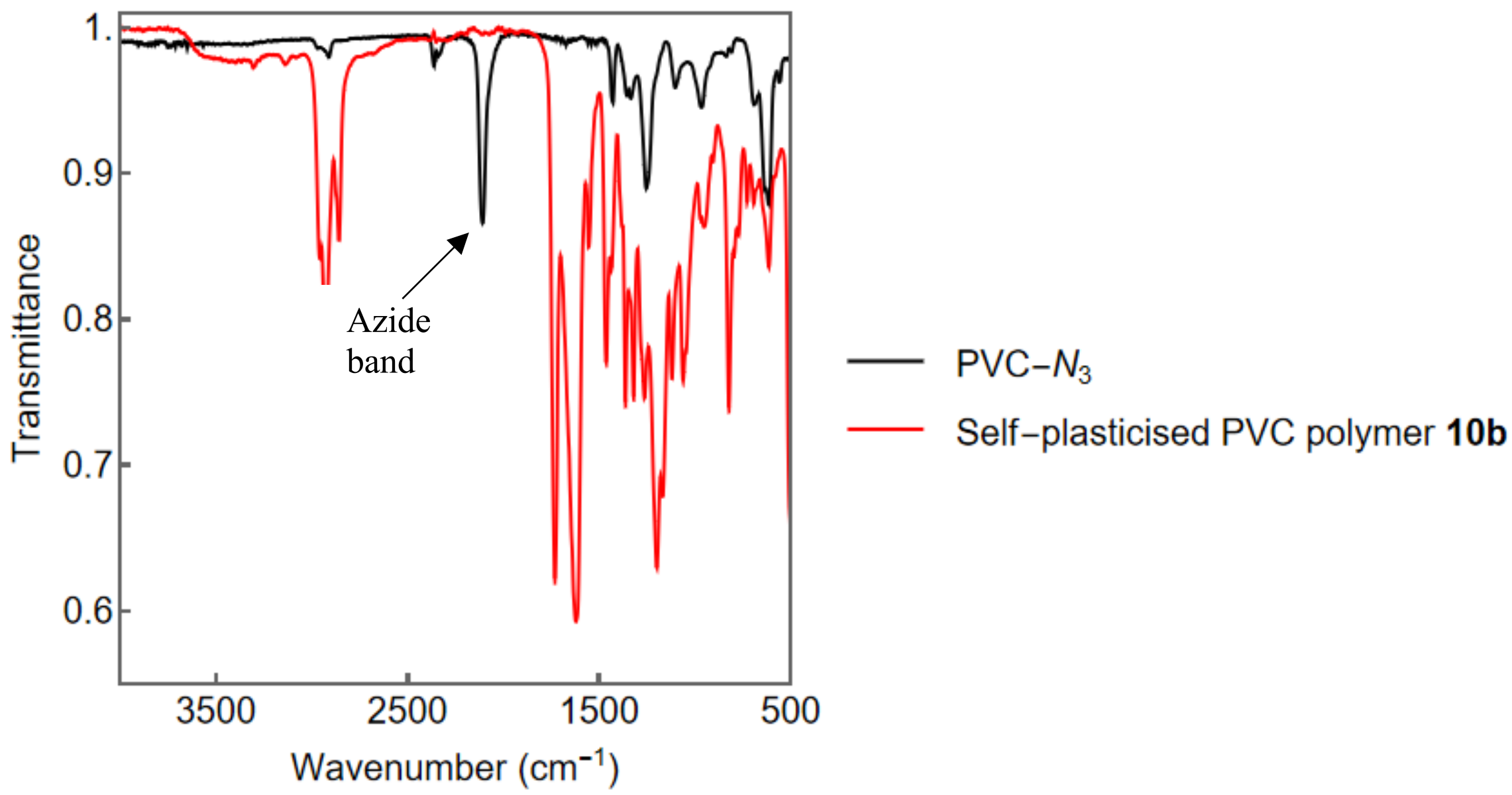

Figure S10: FT-IR spectrum of non-modified PVC (black trace) and self-plasticized PVC polymer 10b (red trace). The final click reaction can be confirmed by the absence of azide groups on the PVC backbone, characterised by the disappearance of the typical azide band present at ~2100 cm^{-1}.

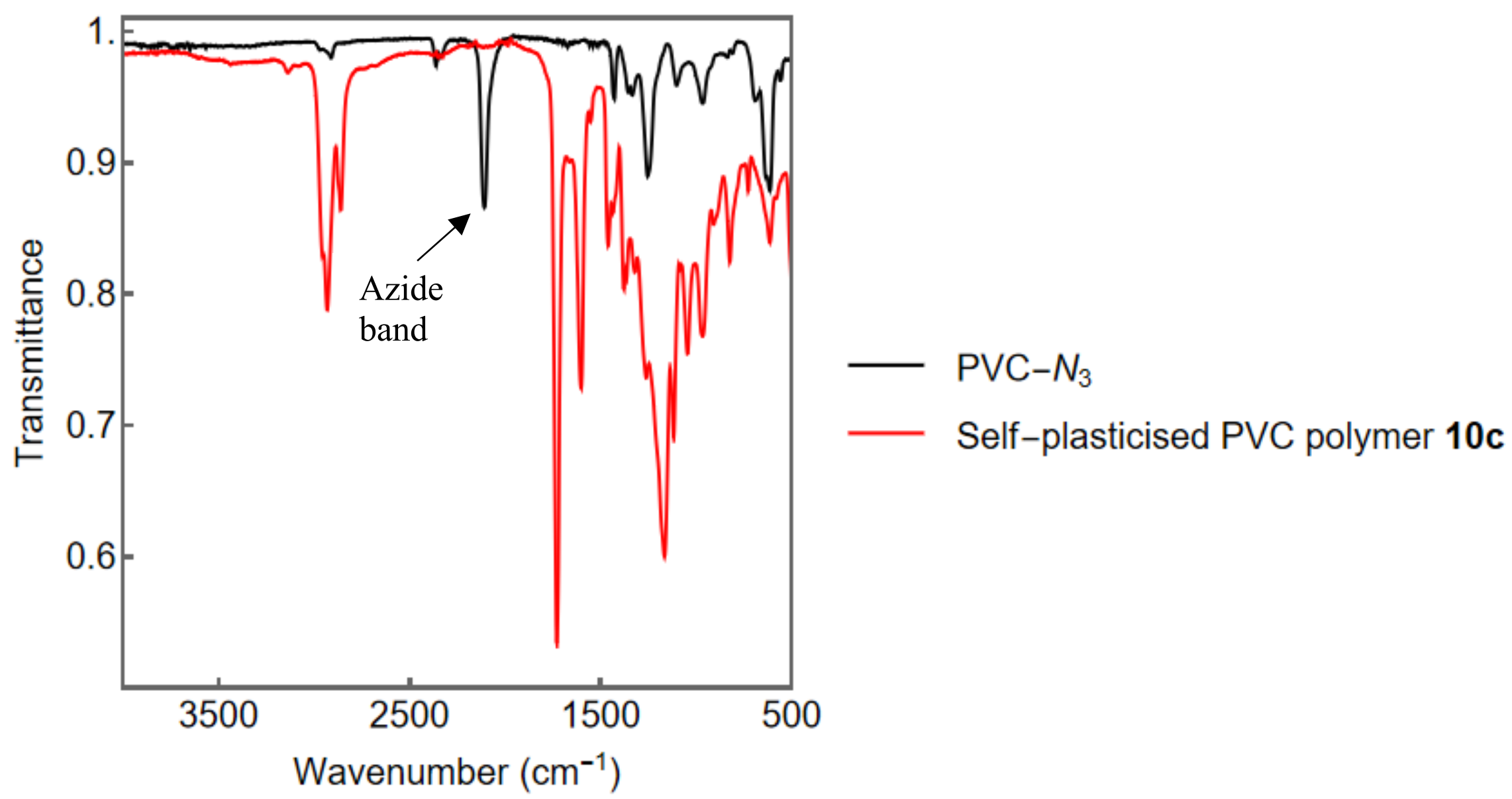

Figure S11: FT-IR spectrum of non-modified PVC (black trace) and self-plasticized PVC polymer 10c (red trace). The final click reaction can be confirmed by the absence of azide groups on the PVC backbone, characterised by the disappearance of the typical azide band present at ~2100 cm^{-1}.

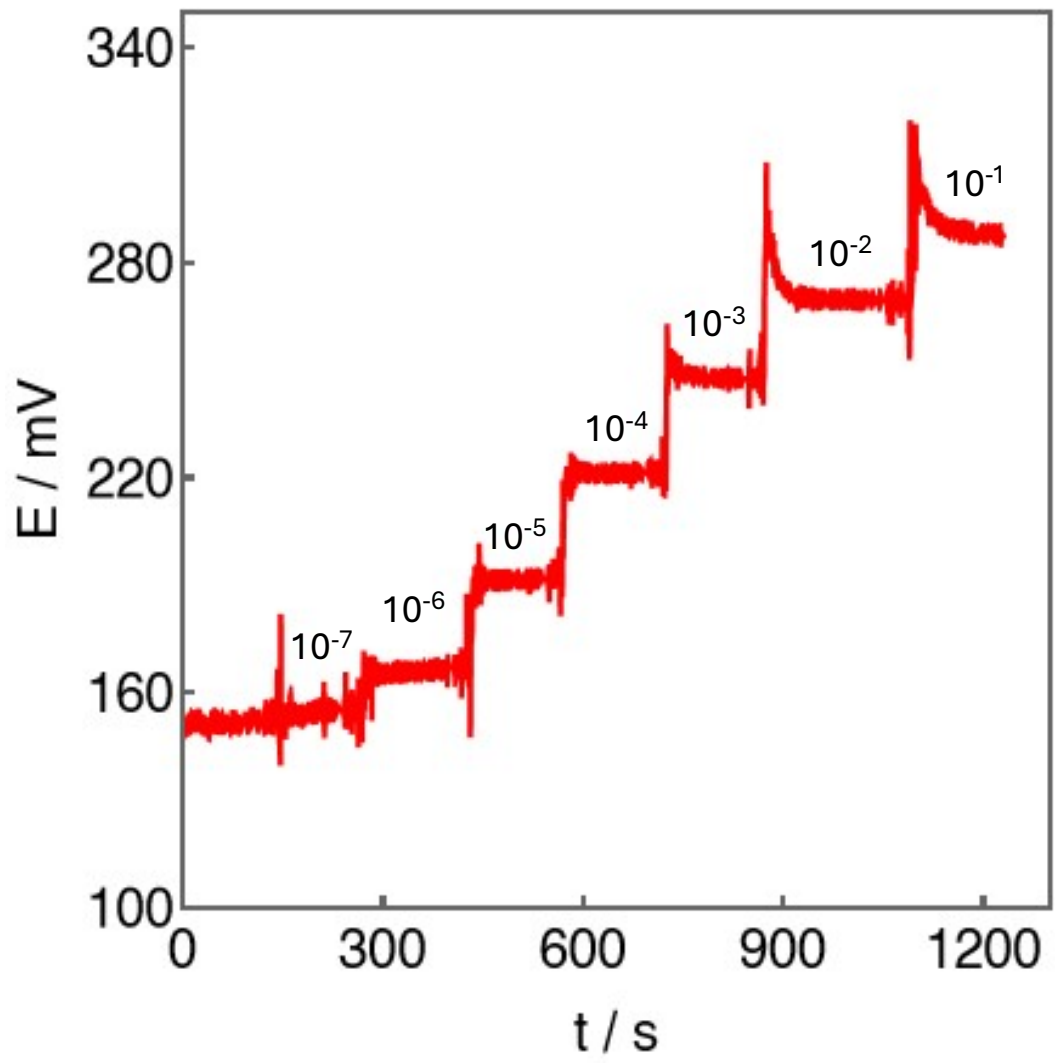

Figure S12: Real time potential response for calcium-selective membrane ranging from concentration 10^{-7} M to 10^{-1} M.

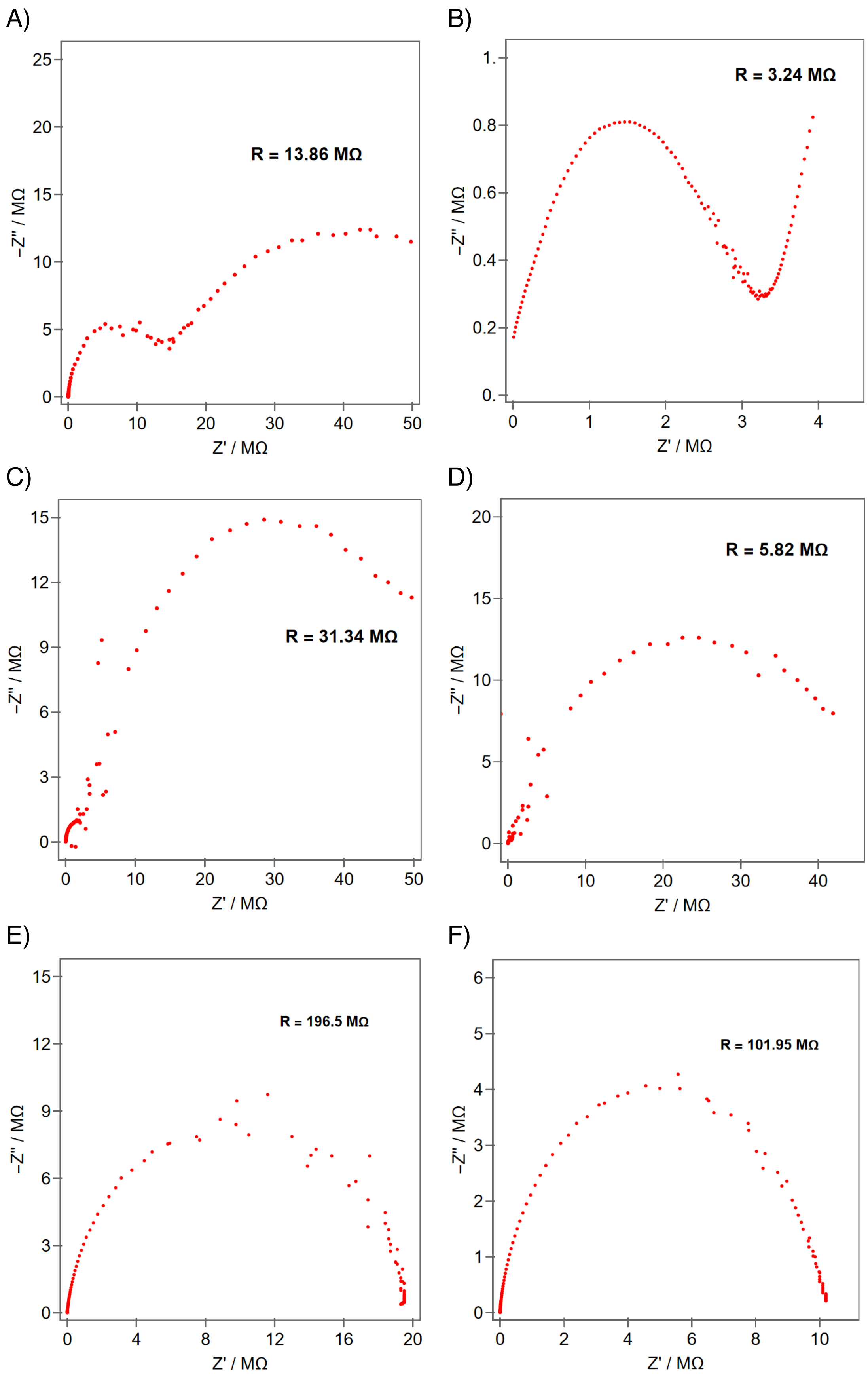
A)
R = 13.86 MΩ
–Z'' / MΩ
Z' / MΩ
B)
R = 3.24 MΩ
–Z'' / MΩ
Z' / MΩ
C)
R = 31.34 MΩ
–Z'' / MΩ
Z' / MΩ
D)
R = 5.82 MΩ
–Z'' / MΩ
Z' / MΩ
E)
R = 196.5 MΩ
–Z'' / MΩ
Z' / MΩ
F)
R = 101.95 MΩ
–Z'' / MΩ
Z' / MΩ

Figure S13: Impedance spectra for A) calcium-selective membrane in 0.1 M $CaCl_2$, B) pH electrode in universal buffer 0.04M pH 7, C) potassium-selective membrane in 0.1 M KCl, D) nitrate-selective electrode in universal buffer 0.1 M KNO_3, E) C_6 linker potassium-selective membrane in 0.1 M KCl, F) C_{10} linker potassium-selective membrane in 0.1 M KCl Reference electrode: double-junction Ag/AgCl/ 3 M KCl/ 1 M LiOAc and counter electrode: Pt rod. Impedance measurements taken at OCP with 100 mV amplitude at a frequency range of 1 MHz to 100 mHz. Resistance estimated using semi-circle fit.

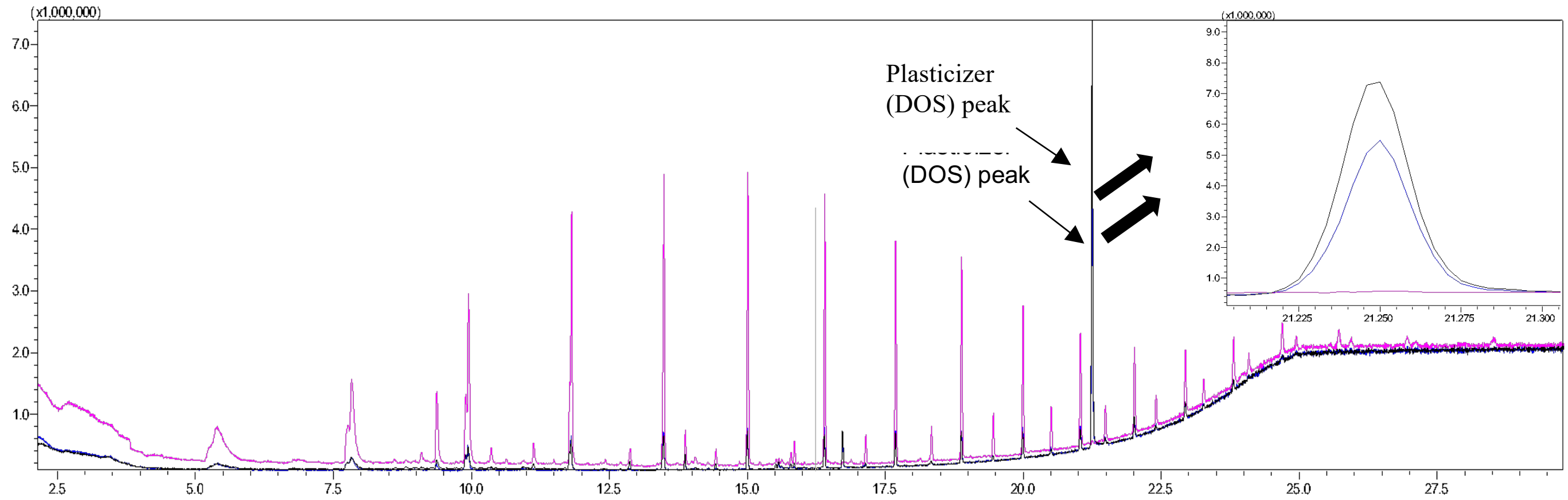

Figure S14: GC-MS spectra of methanol solution of a classical PVC/DOS membrane when exposed to methanol for 4 h. Pink trace - self-plasticized PVC membrane, Blue trace – PVC/DOS membrane, black trace – standard sample of DOS. The tall peak at 21.25 min indicates the plasticizer (DOS) leaching out.
